# Functional and Proteomic Dissection of the Contributions of CodY, SigB and the Hibernation Promoting Factor HPF to Interactions of *Staphylococcus aureus* USA300 with Human Lung Epithelial Cells

**DOI:** 10.1021/acs.jproteome.4c00724

**Published:** 2024-09-20

**Authors:** Xiaofang Li, Larissa M. Busch, Sjouke Piersma, Min Wang, Lei Liu, Manuela Gesell Salazar, Kristin Surmann, Ulrike Mäder, Uwe Völker, Girbe Buist, Jan Maarten van Dijl

**Affiliations:** † Department of Medical Microbiology and Infection Prevention, University of Groningen, University Medical Center Groningen, Hanzeplein 1, 9700 RB Groningen, The Netherlands; ‡ Interfaculty Institute for Genetics and Functional Genomics, Department Functional Genomics, University Medicine Greifswald, D-17475 Greifswald, Germany

**Keywords:** *Staphylococcus aureus*, SigB, CodY, SaHPF, human lung epithelial cells

## Abstract

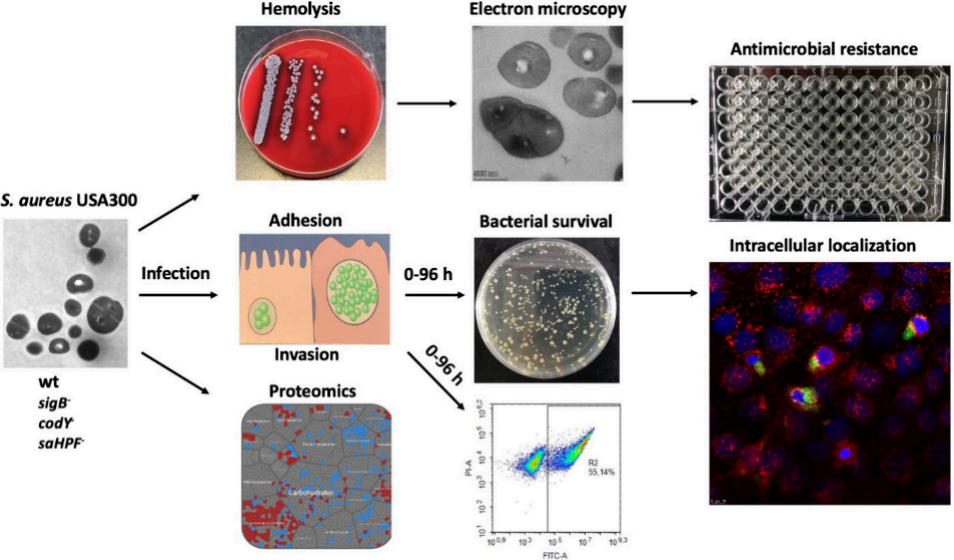

*Staphylococcus aureus* is a leading cause of severe pneumonia. Our recent proteomic investigations into *S. aureus* invasion of human lung epithelial cells revealed three key adaptive responses: activation of the SigB and CodY regulons and upregulation of the hibernation-promoting factor SaHPF. Therefore, our present study aimed at a functional and proteomic dissection of the contributions of CodY, SigB and SaHPF to host invasion using transposon mutants of the methicillin-resistant *S. aureus* USA300. Interestingly, disruption of *codY* resulted in a “small colony variant” phenotype and redirected the bacteria from (phago)lysosomes into the host cell cytoplasm. Furthermore, we show that CodY, SigB and SaHPF contribute differentially to host cell adhesion, invasion, intracellular survival and cytotoxicity. CodY- or SigB-deficient bacteria experienced faster intracellular clearance than the parental strain, underscoring the importance of these regulators for intracellular persistence. We also show an unprecedented role of SaHPF in host cell adhesion and invasion. Proteomic analysis of the different mutants focuses attention on the CodY-perceived metabolic state of the bacteria and the SigB-perceived environmental cues in bacterial decision-making prior and during infection. Additionally, it underscores the impact of the nutritional status and bacterial stress on the initiation and progression of staphylococcal lung infections.

## Introduction

*Staphylococcus aureus* (*S. aureus*) is a frequent cause of human infections, which range from mild skin infections to severe invasive diseases, including bacteremia, sepsis and necrotizing pneumonia. Especially invasive staphylococcal infections are associated with high morbidity and mortality.^1,2^ The clinical introduction of antibiotics, especially β-lactams, has alleviated the harm of *S. aureus* infection to a certain extent in the 20th century.^3^ However, subpopulations of *S. aureus* that became resistant to different antibiotics rapidly emerged, as underscored by the widespread methicillin-resistant *S. aureus* (MRSA).^3^ Infections caused by these resistant lineages are hard to treat with antibiotics, and inadequate therapeutic interventions may lead to chronic infections and relapses. *S. aureus* can acquire antibiotic resistance by changing its genetic makeup through mutations or horizontal transfer of resistance genes from other bacteria.^4,5^ In addition, this bacterium can become less susceptible to antibiotics by metabolic adaptations and by reducing its growth rate. This occurs for instance when *S. aureus* forms biofilms or when it enters the interior of human host cells. Such adaptations lead to subpopulations of so-called persisters, which contribute to chronic and relapsing *S. aureus* infections.^6^

Previous studies have reported that small colony variants (SCVs) of *S. aureus* can give rise to persistent infections.^7−10^ Such SCVs emerge as a slow-growing auxotrophic subpopulation that survives intracellularly over long periods of time.^11,12^ The SCVs derive their name from the smaller colony size, usually about one-tenth of a regular *S. aureus* colony that is observed upon overnight growth on blood agar (BA) plates. In accordance with their low growth rate, the SCVs present enhanced resistance to antibiotics and increased adhesive capabilities, but also a decreased virulence toward host cells, which allows them to persist in the host despite antimicrobial therapy and insults by the innate and adaptive immune systems.^13^ Here it should be noted that, while all persisters can exhibit SCV-like characteristics under particular conditions, not all SCVs are persisters. Specifically, persisters are defined by their transient tolerance to antibiotics due to dormancy or metabolic shutdown, which is reversible. SCVs represent a broader category, which includes phenotypic variants with altered growth and metabolic states, some of which may act as persisters.

Recently, we have investigated the fate of *S. aureus* upon internalization by human lung epithelial cells to better understand the events that lead to staphylococcal pneumonia.^14^ This study involved real-time fluorescence microscopy and electron microscopy to visualize the bacterial behavior upon internalization, as well as detailed proteome profiling to chart the responses of the bacteria to the intracellular host environment and the responses of the host to the internalized bacteria. Over a period of 96 h, we observed the emergence of two distinct bacterial populations. Fast-growing bacteria led to host cell lysis within the first 30 h post infection (p.i.), whereas slow-growing bacteria entered a state of persistence after 48 h p.i.. During the course of infection, we observed a dynamic metabolic crosstalk between the internalized *S. aureus* bacteria and their epithelial host cells, which was attributed to the competition between bacteria and their host for nutrients. Three major adaptive responses of the bacteria were observed. First, upon internalization the bacteria started to highly express the *S. aureus* hibernation-promoting factor (SaHPF). Furthermore, the adaptation to the persister state was accompanied by changes in the abundance of proteins encoded by SigB- and CodY-regulated genes. This suggested that SigB, CodY and SaHPF play crucial roles in the adaptive behavior of *S. aureus* upon internalization.

SaHPF is a 22.2 kDa protein that plays an important role in the formation of 100S ribosome dimers.^15^ This protein is crucial for the repression of translation and, in doing so, it may help *S. aureus* to survive extreme conditions by reducing energy consumption.^16,17^ Accordingly, a *hpf* mutant of *S. aureus* was shown to be attenuated in a murine sepsis model.^18^ Furthermore, Basu et al. reported that the general stress sigma factor SigB and the GTP-responsive transcription factor CodY control the synthesis of SaHPF in response to nutritional and thermal signals.^18^ However, very little is known about the physiological functions of SaHPF. In particular, the possible roles of SaHPF in the adaptation of *S. aureus* to the conditions within host cells, and in intracellular survival, have so far not been investigated.

SigB is an important regulator of the general stress response in *S. aureus*, where it determines the expression of about 200 genes, including genes that are associated with virulence, persistence, cell internalization, membrane transport and antibiotic resistance.^19^ A previous study showed that SigB also contributes to intracellular persistence and that it plays a role in the formation of SCVs in *S. aureus*.^11^ Ishii et al. investigated the effects of pulmonary surfactants on gene expression in *S. aureus* and reported that SigB is important for *S. aureus* adaptation to the lung environment.^20,21^ Others showed that SigB controls the expression of virulence factors involved in biofilm formation and intracellular persistence of *S. aureus* SCVs.^22^

CodY is a global transcriptional repressor and a nutrient-sensing regulator in Gram-positive bacteria. It integrates nutritional signals, such as the levels of branched-chain amino acids (BCAAs) and GTP to modulate metabolic pathways and virulence gene expression.^23^ BCAAs are necessary for protein synthesis, branched-chain fatty acid synthesis, and environmental adaptation in *S. aureus*.^24^ Thus, CodY allows *S. aureus* to adapt its metabolism and virulence in response to the nutritional status of the environment. *S. aureus* CodY is known to regulate over 100 genes, including genes for central carbon metabolism, BCAA metabolism and virulence.^25,26^ In particular, most of the CodY-repressed genes are linked to amino acid metabolism, whereas presumably indirectly CodY-activated genes are linked to nucleotide metabolism or virulence.^27^

Given the devastating pathology of staphylococcal pneumonia, the present study aimed to explore the roles of SaHPF, SigB and CodY in the infectious behavior and survival of *S. aureus* upon internalization by lung epithelial cells. We employed the community-acquired (CA) MRSA strain USA300 for our infection experiments, because CA MRSA is notorious for causing necrotizing pneumonia in the USA, Canada and Europe.^28^ In particular, we used mutant *S. aureus* USA300 derivatives with transposon insertions in *sigB*, *codY* or *saHPF*. Furthermore, we correlated the intracellular behavior of these mutant bacteria with their growth behavior, cell morphology, antibiotic resistance and cellular proteome profile. Interestingly, our findings show that the investigated *codY* mutant bacteria display a SCV-like phenotype that can promote their survival inside human lung epithelial cells.

## Materials and Methods

### Bacterial Strains and Growth Conditions

The investigated *S. aureus* USA300 LAC strain and derivative strains with transposon insertions in the *sigB* (*rpoF*, SAUSA300_2022), *codY* (SAUSA300_1148) or *saHPF* (*yfiA*, SAUSA300_0736) genes are listed in Table 1. The strains were grown overnight at 37 °C in Tryptone Soy Broth (TSB, Oxoid Limited, Hampshire, United Kingdom) with continuous shaking (250 rpm). Although the transposon mutants are resistant to erythromycin, after initial selection of the mutants on erythromycin, whole-genome sequencing showed that there was no need to grow them in the presence of erythromycin for stable maintenance of the transposon (see below). To minimize possible side effects of the presence of erythromycin and for maximal comparability with the wild-type strain, which is erythromycin sensitive, we cultured the bacteria without erythromycin in all experiments. For strains transformed with the plasmid pJL-sar-GFP_redopt-cm, which directs expression of the green fluorescent protein (GFP), the medium was supplemented with 10 μg/mL chloramphenicol (Sigma-Aldrich, Germany) to avoid plasmid loss.

**Table 1:** Bacterial Strains and Plasmid Used in This Study

Strains/plasmid^a^	Genotype/description^b^	Reference
LAC	USA300 wild-type (WT) strain, Em^R^	(29)
NE1109	USA300 JE2, transposon in *sigB*, Em^R^	(30)
NE1555	USA300 JE2, transposon in *codY*, Em^R^	(30)
NE838	USA300 JE2, transposon in *saHPF*, Em^R^	(30)
pJL-sar-GFP_redopt-cm	*Cm*^R^	(31,32)

a NE numbers relate to the strain numbering in the Nebraska transposon mutant library.

b Uniprot accession codes for the *S. aureus* USA300 genes with transposon insertions as indicated. Em^R^, erythromycin resistance. *Cm*^R^, chloramphenicol resistance.

### Genome Sequencing and Bioinformatics

**Figure 1:**
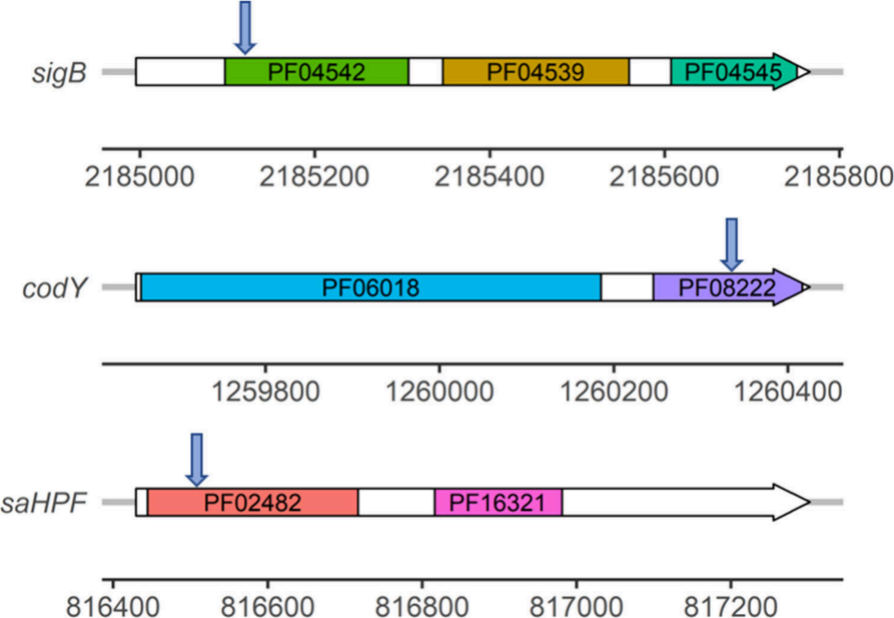
Schematic representation of transposon insertions in the *sigB*, *codY* or *saHPF* genes of *S. aureus* USA300 LAC. The *sigB, codY* and *saHPF* genes are represented by horizontal arrows and transposon insertions are marked by vertical arrows. Color-coded boxes mark the regions encoding domains that have been defined by Pfam (PF04542: Sigma-70 region 2; PF04539: Sigma-70 region 3; PF04545: Sigma-70 region 4; PF06018: CodY GAF-like domain; PF08222: CodY helix-turn-helix domain; PF02482: Sigma 54 modulation protein/S30EA ribosomal protein; PF16321: Sigma 54 modulation/S30EA ribosomal protein C terminus). The nucleotide coordinates in the genome of *S. aureus* USA300 LAC are indicated below each gene.

To verify the correct insertion sites of the *bursa aurealis* transposon in the *sigB*, *codY* or *saHPF* mutant bacteria, whole-genome paired-end sequencing was performed by 500 cycles on a MiSeq Sequencer using the MiSeq Reagent Kit v2 (Illumina). De novo genome assembly was performed using SPAdes 3.13.1 and the genome sequence of the *S. aureus* USA300 LAC strain was used as a reference. The sites of transposon insertion in the genes are schematically indicated in Figure 1.

### Plasmid Isolation and Transformation

All strains were transformed with plasmid pJL-sar-GFP_redopt-cm to express a codon-optimized GFP as described previously.^32,33^ The plasmid DNA was isolated from *S. aureus* RN4220 using a plasmid isolation kit from AnalytikJena. To this end, the bacteria were collected from an overnight culture in TSB by centrifugation for 5 min at 13,400 g. The isolated plasmid DNA was verified by agarose gel electrophoresis and stored at −20 °C.

Preparation of competent *S. aureus* USA300 cells and electroporation were done as described previously^34^ with minor adjustments. In short, the *S. aureus* USA300 LAC strain and its isogenic *sigB*, *codY* or *saHPF* mutants were grown overnight in TSB medium. Subsequently, the cultures were diluted to an optical density at 600 nm (OD_600_) of 0.05 with fresh TSB at a final volume of 50 mL. The cultures were then incubated in a water bath at 37 °C with 150 rpm shaking until they reached the exponential growth phase (OD_600_ 0.5–0.6). The cultures were chilled on ice for 30 min, bacterial cells were collected by centrifugation for 10 min (6000 g, 4 °C), and the supernatant was removed. The pellet was resuspended in 50 mL of precooled 0.5 M sucrose solution and centrifuged (6000 g, 4 °C). This washing step was repeated 3 times and then the pellet was resuspended in 250 μL precooled 0.5 M sucrose solution. Lastly, aliquots of 50 μL were frozen immediately by using liquid nitrogen and stored at −80 °C for future use. For electroporation, the competent cells were thawed on ice for 5 min, and 50 μL of the cells were then mixed with 1–4 μg of plasmid DNA in a chilled 2 mm electroporation cuvette (BioRad, California, USA). The electroporation was performed using a Gene Pulser Xcell Electroporation Systems (BioRad, California, USA) at the following settings: 2500 V, 200 Ω and 25 μF. The transformed cells were then immediately mixed with 1 mL prewarmed TSB and incubated for 2 h at 37 °C with shaking at 250 rpm. Lastly, the cells were plated on tryptic soy agar (TSA) containing 10 μg/mL chloramphenicol and incubated overnight at 37 °C to select for the presence of pJL-sar-GFP_redopt-cm. Transformants were checked for GFP-expression using a Leica DM5500 B florescence microscope (Leica Biosystems, Germany), and they were verified by mass spectrometry using a MALDI Biotyper (Bruker Daltonik, Bremen, Germany).^35^ The transformed bacteria were cultured in TSB medium with 10 μg/mL chloramphenicol and stored in 20% glycerol at −80 °C.

### Phenotypic Characterization of Mutant Bacteria

For phenotypic analysis with respect to colony size, color and hemolysin production, the bacteria were plated on blood agar (BA) containing 5% sheep blood. To monitor and compare bacterial growth, the different strains were cultured as previously described.^36^ In brief, the bacteria were grown overnight in TSB. On the next day, the bacteria were diluted to an OD_600_ of 0.06 in Roswell Park Memorial Institute 1640 medium (RPMI; Gibco, New York) supplemented with 2 mM l-glutamine (Thermo Fisher Scientific, Waltham, USA) and 10 μg/mL chloramphenicol, and then precultured in a water bath at 37 °C with shaking (150 rpm). Notably, the RPMI medium was selected for our experiments, because our previous genome-wide transcript profiling experiments have shown that the transcriptome of *S. aureus* grown on RPMI closely resembles the transcriptome of *S. aureus* grown in human plasma.^37^ This implies that RPMI medium closely mimics the (iron-restricted) nutritional conditions encountered by *S. aureus* during an invasive infection. When the OD_600_ reached 0.5, the cultures were diluted to an OD_600_ of 0.06 with fresh RPMI, and culturing was continued in the water bath under the same conditions to ensure that the bacteria were in the same physiological state and fully adapted to the RPMI medium. Growth curves were recorded by measuring the OD_600_ using a Cary 60 UV–vis spectrophotometer (Agilent Technologies, USA) over a period of 24 h. The OD_600_ was measured in triplicate every hour for the first 12 h and after 24 h of growth.

To quantify the number of viable bacteria, the strains were cultured in RPMI medium as described above.^36^ When the OD_600_ reached 0.5, the cultures were serially diluted in sterile phosphate-buffered saline (PBS; pH 7.2) and plated on TSA with 10 μg/mL chloramphenicol. Upon incubation for 16 to 20 h at 37 °C, the numbers of colony-forming units (CFUs) were counted. Each measurement was performed in triplicate and the experiments were repeated three times.

### Antimicrobial Susceptibility Tests

Antimicrobial susceptibility tests were performed using a VITEK 2 system (bioMérieux, Inc., Durham, NC) and AST-P657 cards according to the manufacturer’s instructions. With these cards, the susceptibility for cefoxitin, benzylpenicillin, oxacillin, gentamicin, tobramycin, ciprofloxacin, clindamycin, erythromycin, clindamycin, linezolid, teicoplanin, vancomycin, tetracycline, fosfomycin, nitrofurantoin, fusidic acid, mupirocin, rifampicin and trimethoprim/sulfamethoxazole was tested. The results obtained from the VITEK analyses were validated using the Advanced Expert System following the protocols of the Clinical and Laboratory Standards Institute (CLSI, https://clsi.org/). Next, the minimum inhibitory concentrations (MIC) of benzylpenicillin, nitrofurantoin and vancomycin were determined by serial (2-fold) dilution in Mueller Hinton (MH) broth. The MIC values here represent the lowest concentration of antibiotics that completely inhibited the growth of the strains upon overnight culture. The tested concentrations of benzylpenicillin ranged from 1 to 32 μg/mL, nitrofurantoin concentrations ranged from 2 to 128 μg/mL, and vancomycin concentrations ranged from 0.5 to 8 μg/mL. The antimicrobial susceptibility tests were performed in triplicate and repeated three times.

### Live/Dead Viability Tests

The bacterial viability testing was performed using the live/dead baclight bacterial viability kit (L7012, Thermo Fisher Scientific) in accordance with the manufacturer’s instructions. In brief, the bacteria were cultured overnight in MH broth and, on the next day, the bacteria were diluted to an OD_600_ of 0.06 in fresh MH broth and incubated in a shaking water bath (150 rpm) at 37 °C until an OD_600_ of 0.5. The bacteria were then treated with 4 × MIC of vancomycin for 3 h. For control, live- and ethanol-killed bacteria were mixed to achieve various proportions of live:dead bacteria. A reagent mixture of propidium iodide and SYTO 9 was added to the bacterial test and control samples and then the bacteria were incubated at RT in the dark for 15 min. Live and dead bacteria were quantified using a NovoCyte Quanteon flow cytometer (Agilent, The Netherlands).

### Cell Line and Cultivation

The transformed human bronchial epithelial cell line 16HBE14o- was used for infection experiments.^38^ The cells were cultured in eukaryotic minimal essential medium (eMEM; Biochrom AG, Berlin, Germany) supplemented with 10% (v/v) fetal calf serum (FCS; Biochrom AG), 1% (v/v) sodium pyruvate (Sigma-Aldrich, Germany) and 1% (v/v) nonessential amino acids (PAN-Biotech GmbH) at 37 °C with 5% CO_2_. The medium was changed every 2 days and the cells were split every 3 days with 0.25% trypsin-EDTA (Gibco, Grand Island, NY). All cells used in this project underwent less than 10 passages to avoid loss of primary cell characteristics.

### Bacterial Association and Internalization in Human Lung Epithelial Cells

Bacterial internalization experiments were performed using a confluent layer of 16HBE14o- cells as described previously.^14^ Briefly, 1 × 10^5^ cells per well were seeded in 12-well plates 3 days prior to infection. On the day of infection, overnight cultured bacteria were used to inoculate fresh RPMI medium as previously described^36^ to obtain cultures in the exponential phase. Next, the lung epithelial cell number in one well was counted and a multiplicity of infection (MOI) of 100 was used for the adhesion and internalization experiments. To this end, the bacteria were diluted in eMEM cell culture medium to achieve the MOI of 100 based on the CFU counting results described above and, subsequently, the eMEM with the bacteria was used to replace the cell culture medium. Cell culture was then continued for 1 h at 37 °C with 5% CO_2_.

To determine the number of internalized bacteria, the medium was removed and the cells were washed once with PBS. Fresh eMEM medium containing 25 μg/mL lysostaphin (AMBI Products, New York) was then added to the cells. Lysostaphin was included in the medium to kill bacteria adhering to the surface of the cells, and to prevent reinfection of the cells by bacteria that had been internalized but subsequently escaped by host cell lysis. After incubation at 37 °C for 30 min, the medium with lysostaphin was removed, and the cells were washed once with PBS. The cells were then lysed by treating them with cold Milli-Q water for 15 min.

To determine the number of adherent bacteria, the medium was removed, the epithelial cells were washed with PBS and then treated with cold Milli-Q water for 15 min. The internalized and attached bacteria were collected after cell lysis, the samples were then serially diluted in PBS and plated on TSA plates supplemented with 10 μg/mL chloramphenicol in triplicate for “total CFU-counting”. It should be noted that, following this procedure, the measured “total CFU counts” reflected both the adherent (not yet internalized) and the internalized bacteria. To determine the number of adherent bacteria in each sample, we subtracted the respective CFU count of internalized bacteria that was determined as described in the previous paragraph, from the “total CFU count”. The experiments were repeated three times.

### Flow Cytometric Counting of Lung Epithelial Cells and Internalized Bacteria

Flow cytometry was used to count the 16HBE14o- cells and internalized bacteria at 0 h, 2 h, 7 h, 24 h, 48 h, 72 and 96 h p.i.. After infection with bacteria for 1 h and incubation in medium containing lysostaphin, two groups of samples were collected in triplicate at the different time points. One sample group was used for counting the cells upon treatment with 0.25% trypsin-EDTA. The other sample group was used for counting the internalized bacteria upon treatment of the cells with 0.05% sodium dodecyl sulfate (SDS) in PBS for 5 min. The infected human cells and internalized bacteria were then counted using a NovoCyte Quanteon flow cytometer, where GFP was excited with a 488 nm laser and the emitted fluorescence was detected at 525/40 nm. All experiments were repeated three times, and the flow cytometry data was analyzed by FCS Express software (De novo Software, USA).

### Confocal Fluorescence Microscopy

To investigate the subcellular colocalization of the lysosomal-associated membrane protein1 (LAMP-1) and internalized bacteria at different time points, immunofluorescence microscopy was performed using a Leica TCS SP8 confocal laser scanning microscope (Leica Microsystems, Wetzlar, Germany). Autoclaved coverslips were placed in 12-well plates (Greiner Bio-One, Austria) and 1 × 10^5^ cells per well were seeded 3 days prior to the experiment. On the day of the immunofluorescence microscopy, the internalization experiments were performed as described above. After incubation with lysostaphin, the coverslips were collected at different time points and the cells were fixed with 4% paraformaldehyde for 20 min at room temperature (RT). Next, the cells were permeabilized with 0.5% Tween-20 for 30 min at RT and then blocked with 2% bovine serum albumin (BSA; Sigma-Aldrich, USA) and 10% normal goat serum (Thermo Fisher Scientific, USA) in PBS overnight at 4 °C. All antibodies listed below were diluted in this blocking solution. Next day, additional blocking was performed by incubating with a polyclonal rabbit antibody against TrxA of *S. aureus*(39) (1:5000) for 2 h at RT in a humidified chamber. Subsequently, a primary mouse antibody (CD107a; BD, United States) was used to determine the subcellular localization of LAMP-1 at a dilution of 1:100. Upon incubation for 2 h at RT in a humidified chamber, a goat antimouse secondary antibody conjugated with Alexa Fluor 594 (Invitrogen, Netherlands) was added at a 1:500 dilution and incubation was continued for 1 h at RT. Lastly, 4′, 6-diamidino2-phenylindole (DAPI; Roche, Switzerland) was used to stain the nuclear DNA, the slides were mounted with Mowiol 4–88 (Merk Millipore, USA) and stored at −20 °C until microscopic visualization.

### Transmission Electron Microscopy

Transmission electron microscopy (TEM) was used to study the morphology of the bacterial cells. Bacteria were grown to the exponential phase in RPMI and then collected by centrifugation at 3000 rpm for 5 min. Subsequently, the supernatant was discarded, and the cells were fixed with 2% glutaraldehyde and 2% paraformaldehyde in 0.2 M sodium cacodylate buffer (pH 7.4) for 30 min at RT. The bacteria were then pelleted by centrifugation and the supernatant was discarded. The pelleted bacteria were embedded in 200 μL 2% low-melting point agarose (Sigma-Aldrich, St. Luis, USA) in 0.1 M sodium cacodylate buffer. Upon solidification of the agarose, the embedded samples were cut into small pieces (around 2 × 2 × 2 mm), which were washed 3 times for 5 min with 0.1 M cacodylate buffer at RT. Postfixation was performed with 1% osmium tetroxide and 1.5% potassium ferrocyanide in 0.1 M sodium cacodylate at 4 °C for 2 h. The samples thus obtained were washed with Milli-Q water and stepwise dehydrated by subsequent incubations in ethanol solutions of 30%, 50%, 70% and 100%. The last dehydration step in absolute ethanol involved 4 incubations of 30 min. Lastly, the samples were embedded in EPON resin, which was allowed to polymerize overnight at 37 °C, and then incubated at 58 °C for another night. After polymerization, the samples were cut into ultrathin sections (100 nm) using a UC7 ultramicrotome (Leica, Vienna, Austria) and counterstained with 4% neodymium. Images were recorded with a Talos F200X G2 TEM (Thermo Fisher Scientific) operated at 80 kV. Images were analyzed using FIJI software (https://fiji.sc/).

### LDH Cytotoxicity Assays

To assess the cytotoxicity of the bacteria toward 16HBE14o- cells, the LDH cytotoxicity assay kit was used according to the manufacturer’s instructions (Thermo Fisher Scientific). Before the experiments, the optimum cell number was determined (1 × 10^4^). For the main experiment, aliquots of 1 × 10^4^ cells were resuspended in 100 μL eMEM cell culture medium and seeded in triplicate wells in a 96-well tissue culture plate followed by incubation overnight at 37 °C with 5% CO_2_. The bacteria were cultured overnight in TSB. On the day of the experiment, the overnight cultured bacteria were diluted to an OD_600_ of 0.06 with RPMI medium and culturing was continued in a shaking water bath (150 rpm) until the exponential phase was reached (OD_600_ ∼ 0.5). Next, the bacteria were diluted again to an OD_600_ of 0.06 with RPMI medium, and culturing was again continued to the exponential phase to ensure that the bacteria were in the same physiological condition and fully adapted to the RPMI medium. To test the cytotoxicity of the bacteria toward epithelial cells using the LDH activity test, 10 μL aliquots of bacteria (MOI 100) were added to each set of triplicate wells of cells. Two groups of controls were performed. To test for spontaneous LDH release, 10 μL of sterile water was added to one set of triplicate wells with cells. To measure the maximal LDH activity, 10 μL of 10x lysis buffer (1x in final concentration) was added to another set of triplicate wells with cells. The plate was then incubated at 37 °C with 5% CO_2_ for 1 h. Subsequently, the samples were transferred to a new flat bottom 96-well plate and 50 μL of reaction mixture was added to each sample well, which was followed by incubation for 30 min at RT protected from light. Lastly 50 μL of stop solution was added to each sample well and the absorbance at 490 and 680 nm was measured. To determine LDH activity, the 680 nm absorbance value (background) was subtracted from the 490 nm absorbance value. The percentage of cytotoxicity was calculated by using the following formula: % cytotoxicity = [(Bacteria-treated LDH activity–spontaneous LDH activity)/(maximum LDH activity- spontaneous LDH activity)] × 100.

### Sample Harvesting and Preparation for Mass Spectrometric Analysis

*S. aureus* USA300 and the *sigB*, *codY*, or *saHPF* mutant bacteria were grown overnight at 37 °C in TSB with vigorous shaking (250 rpm). On the next day, the overnight-cultured bacteria were diluted to an OD_600_ of 0.06 with RPMI medium and culturing was continued in a shaking water bath (150 rpm) until the exponential phase was reached (OD_600_ ∼ 0.5). Next, the bacteria were diluted again into 50 mL of fresh prewarmed RPMI medium to a final OD_600_ of 0.06, and the bacterial cultivation was continued until an OD_600_ of ±0.5 was reached. At this stage, 20 mL culture samples for proteome analysis of exponentially growing bacteria were collected. For the proteome analysis of bacteria that were about 2 h in the postexponential growth phase (for convenience here referred to as the “stationary growth phase”), the cultivation of the WT USA300 bacteria and the *sigB* or *saHPF* mutant bacteria was continued for an additional 5 h and culture samples of 10 mL were collected. To obtain samples of the slower growing *codY* mutant bacteria at 2 h in the postexponential (“stationary”) growth phase, cultivation was continued for 6 h (Supplemental Figure S1). Upon harvesting of the cells, the amount of OD units was adjusted to ensure that approximate equal numbers of exponential and stationary phase bacteria were used for the subsequent cell disruption and protein extraction. The collected samples were immediately snap-frozen in liquid nitrogen followed by inversion of the sample tubes, and this was repeated three times until the samples were cooled on ice. The bacterial cells were then separated from the growth medium by centrifugation at 4 °C (16000g, 10 min). The bacterial cells were washed once with ice-cold 20 mM HEPES (4-(2-hydroxyethyl)-1-piperazineethanesulfonic acid) buffer (pH 8.0) and then the pellet was resuspended in 1 mL fresh HEPES buffer and transferred to a 2-ml reaction tube. Lastly, the bacterial cells were again collected by centrifugation at 4 °C (16000g, 3 min), the supernatant was discarded, the pellet was frozen in liquid nitrogen, and stored at −80 °C until use.

Frozen bacterial cells were mechanically disrupted using a bead mill (Retsch GmbH, Haan, Germany; 3 min, 2600 rpm) and the cell powder was resuspended in 20 mM HEPES + 1% SDS. For degradation of nucleic acids, cell lysates were treated with benzonase (Pierce, Thermo Fisher Scientific, MA, USA; 2.5 U, 4 mM MgCl2, 20 min, 37 °C) and ultrasonicated for 5 min in an ultrasonic bath (Sonorex, Bandelin, Germany). Cell debris were removed by centrifugation (30 min, 17,000 g, RT). Protein concentrations were determined using a Micro BCA Protein Assay Kit (Pierce, Thermo Fisher Scientific, MA, USA).

Tryptic digestion of proteins and peptide purification for mass spectrometry (MS) were performed by single-pot solid-phase-enhanced sample preparation (SP3) as described by Blankenburg et al.^40^ Briefly, 5 μg protein mixture per sample were incubated with 100 μg of hydrophilic (GE Healthcare, Little Chalfont, UK) and hydrophobic (Thermo Fisher Scientific, MA, USA) carboxylate-modified magnetic SeraMag Speed Beads in 70% acetonitrile (ACN) (200 g, RT, 18 min). Then, the beads were washed twice with 70% ethanol and once with 100% ACN. For digestion, the beads were incubated with 200 ng trypsin (Promega Corporation, Wisconsin, USA) in ammonium-bicarbonate buffer (37 °C, 16h) and the digestion was stopped by incubation in 95% ACN (18 min, 200 g, RT). The beads were washed with 100% ACN and peptides were eluted from the beads with 2% dimethyl sulfoxide and by ultrasonication (5 min).

### Mass Spectrometric Measurements and Data Analysis

Peptides were suspended in buffer A (4% ACN, 0.2% acetic acid). For LC-MS/MS analyses, separation of tryptic peptide solutions was carried out on an Ultimate 3000 nano-LC system (Thermo Fisher Scientific, MA, USA) and the peptides were analyzed in data-independent acquisition (DIA) mode on a Q Exactive HF mass spectrometer (Thermo Fisher Scientific, MA, USA). One biological replicate of each individual sample was collected and analyzed. Furthermore, each sample was injected as three technical replicates of 6 μL each. For further details see Supplemental Tables S1A and S1B.

DIA MS data was searched against the USA300 FPR3757 reference strain protein database (fasta file downloaded 22.09.22 from AureoWiki^41^ comprising 2917 staphylococcal proteins using the Spectronaut software (v16.2.220903.53000; Biognosys AG, Schlieren, Switzerland) with settings described in Supplemental Table S1C in the direct DIA analysis type in accordance with the procedure described by Michalik et al.^42^

Outlier samples were removed manually from the data set and the global median-normalized Spectronaut-processed data was further analyzed in R (v4.1.2). All R packages used for analysis and visualization are listed in Supplemental Table S1D. For global proteome analysis, proteins identified with at least two peptides were considered. Peptide-based ROPECA^43^ statistics were calculated protein-wise for each mutant compared to the USA300 WT strain. Protein levels were considered to differ significantly between a mutant and the USA300 WT if the fdr-adjusted p-value (q-value) was smaller than 0.05 and the absolute fold change was at least 1.5. The mass spectrometry proteomics data have been deposited to the ProteomeXchange Consortium via the PRIDE^44^ partner repository with the data set identifier PXD044962. The results are summarized in Supplemental Table S2.

For Fisher Exact-based enrichment analyses, RegPrecise-based^45^ regulon information from AureoWiki, corresponding to the genes of *S. aureus* NCTC8325, was mapped to the USA300 FPR3757 genes according to the pan genome orthologue information in AureoWiki (downloaded 21.09.22).

Voronoi treemaps were made using the R package WeightedTreemaps (https://github.com/m-jahn/WeightedTreemaps) and the AureoWiki database for TheSEED^46^ functional categories from USA300 FPR3757 (NCBI,UniProt 2013–06–10).

## Results

### Phenotypic Differences between *S. aureus* USA300 and the *sigB*, *codY* or *saHPF* Mutant Bacteria

**Figure 2:**
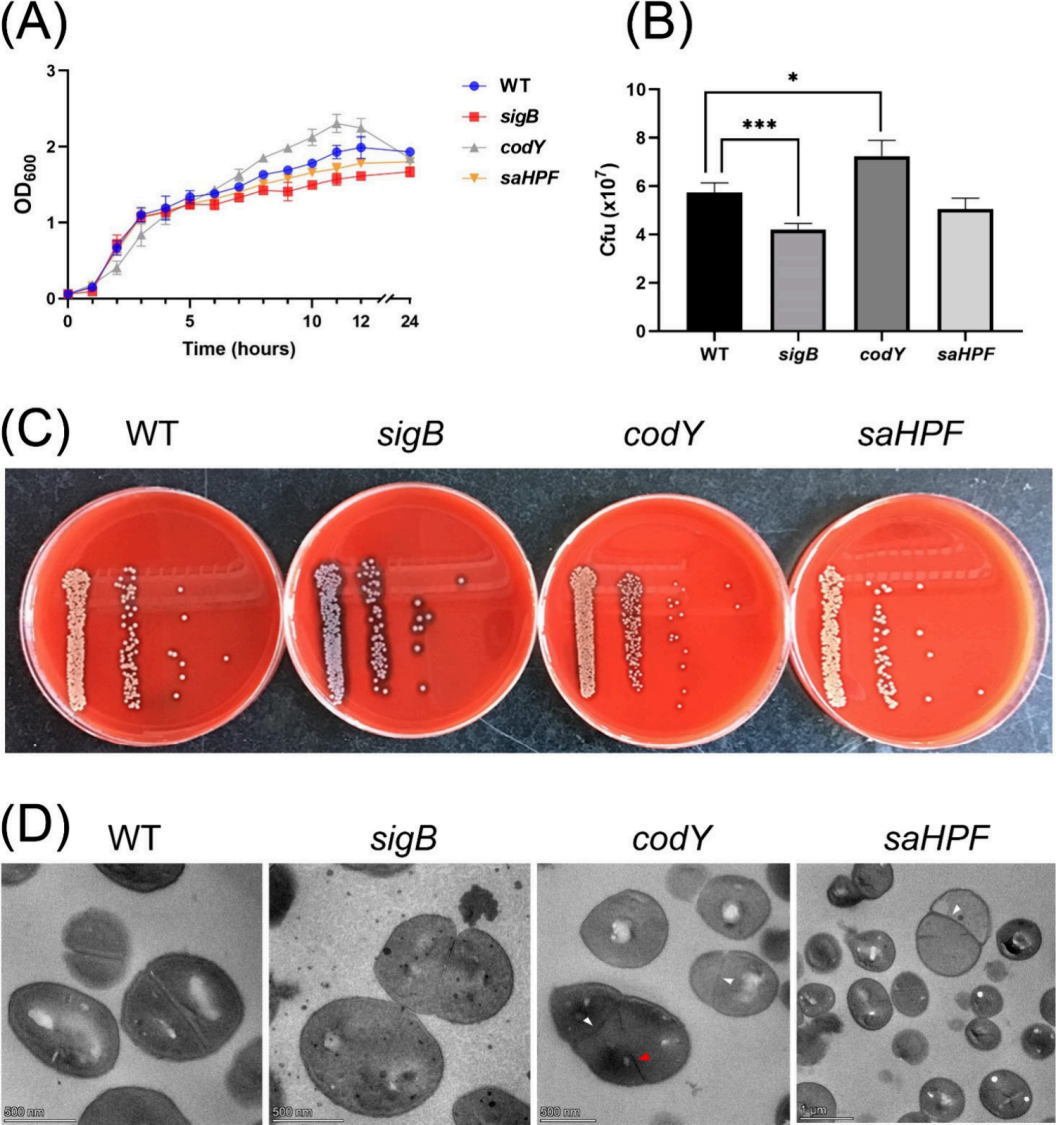
Growth analysis of *S. aureus* USA300 WT and the *sigB, codY,* or *saHPF* mutant strains. (A) Average growth curves of three independent measurements of the bacterial strains in shaken RPMI cultures. (B) CFU counts of bacteria cultured in RPMI to an OD_600_ of 0.5. The statistical significance was assessed by two-tailed *t* tests. A P-value <0.05 was considered significant. CFU counts of the mutants that are statistically different from the WT strain are marked (*, *p* < 0.05; ***, *p* < 0.005). (C) Colony formation on BA plates after overnight growth. (D) TEM images of the WT and mutant bacteria grown to the midexponential growth phase in RPMI. Observed septation defects in cell morphology are indicated with white arrowheads. A ring-like structure in a *codY* mutant bacterium, which may be caused by mislocation of the midcell divisome to the peripheral wall instead of the septum, is indicated with a red arrowhead. The scale bars in the images of the WT, *sigB* and *codY* strains represent 500 nm, the scale bar in the image of the *saHPF* strain represents 1 μm.

To compare the growth behavior of the *S. aureus* USA300 WT strain and its *sigB*, *codY* and *saHPF* transposon mutants, the different strains were grown in RPMI and growth was followed by measuring the OD_600_. As shown in Figure 2A, the *codY* mutant bacteria grew slower than the WT bacteria in the exponential phase but reached a higher OD_600_ in the stationary phase. In contrast, the *sigB* and *saHPF* mutant bacteria reached a slightly lower OD_600_ in the stationary phase compared to the WT strain but displayed the same growth characteristics in exponential phase. Furthermore, the phenotypes of the different strains were inspected upon plating on blood agar (BA). Interestingly, the strains displayed different colony phenotypes. A decreased yellow pigmentation and increased hemolysis were observed for colonies of the *sigB* mutant (Figure 2C). In addition, colonies of the *codY* mutant had on average a 1.4-fold smaller Feret’s diameter than those of the WT strain upon overnight incubation. On the other hand, colonies of the *sigB* and *saHPF* mutants were slightly larger than those of the WT strain. Furthermore, culture samples collected at an OD_600_ of 0.5 showed differences in CFU counts, which is suggestive of differences in the bacterial cell morphology (Figure 2B). In particular, the *codY* mutant showed higher CFU counts per OD_600_ compared to the WT strain, whereas the *sigB* or *saHPF* mutants showed lower CFU counts per OD_600_ compared to the WT strain. Lastly, TEM was applied to correlate the results from the growth experiments with bacterial morphology at the level of individual cells. As expected, the septa of cells from the WT strain were found to be located in the middle of the dividing bacteria. In contrast, the *codY* or *saHPF* mutant bacteria showed an aberrant septation (Figure 2D). Furthermore, the *codY* mutant bacteria displayed multiple ring-like structures, which may be caused by mislocation of the midcell divisome to the peripheral wall instead of the septum. These results show that the different mutations have significant effects on the bacterial growth and cell morphology, with the *codY* mutant bacteria showing a SCV-like phenotype.

### Altered Antimicrobial Susceptibility of the *sigB*, *codY* or *saHPF* Mutant Strains

To investigate whether the altered morphology of *sigB*, *codY* or *saHPF* mutant bacteria can be correlated with an altered susceptibility to antibiotics, the VITEK 2 system and AST-P657 cards were used. With the AST-P657 cards the minimal inhibitory concentrations (MIC) of 20 different antibiotics can be monitored. Indeed, differences were observed in the resistance of the different strains against benzylpenicillin, vancomycin and nitrofurantoin, whereas no differences were observed in the resistance against the 17 other antibiotics on the card. Serial broth dilution assays were subsequently performed to verify the strains’ MICs for benzylpenicillin, vancomycin and nitrofurantoin (Table 2). This showed that the *codY* mutant is more resistant to benzylpenicillin, vancomycin and nitrofurantoin compared to the WT strain, and the *codY* mutant was also more resistant to benzylpenicillin and vancomycin than the *sigB* and *saHPF* mutants. In contrast, the *sigB* and *saHPF* mutants presented a decreased resistance to benzylpenicillin compared to the WT strain. Notably, the MIC of benzylpenicillin determined for the *saHPF* mutant was 3-fold lower than the MIC determined for the WT strain. Taken together, these results show that the three mutant strains display altered antimicrobial susceptibility toward the cell wall-active antibiotics benzylpenicillin and/or vancomycin, which is in line with the altered septation of these strains. Furthermore, the increased resistance of the *codY* mutant toward all three tested antibiotics is in accordance with its slower growth rate, since these antibiotics mostly affect growing bacteria.

**Table 2:** Minimum Inhibitory Concentrations (MIC) of Benzylpenicillin, Vancomycin and Nitrofurantoin for the Strains

	MIC (μg/mL)^a^		
Strains	Benzylpenicillin	Vancomycin	Nitrofurantoin
WT	8	2	16
*sigB*	4	2	32
*codY*	16	4	32
*saHPF*	1	2	32

a The MIC of each antibiotic was determined in triplicate with at least two biological replicates by serial microdilution in Muller Hinton broth. The mean values of the MIC are indicated.

To verify the increased resistance of the *codY* mutant strain toward vancomycin, the different bacterial strains were grown for 3 h in the presence of 4 times the MIC measured for this antibiotic. The percentage of surviving bacteria was then measured using live/dead staining and flow cytometry (Table 3). The results show that, indeed, the *codY* mutant bacteria were more resistant to vancomycin than the other strains.

**Table 3:** Live/Dead Viability Test by Flow Cytometry after Treatment with Vancomycin

Strains	Live bacteria (%)^a^
WT	37.7 ± 4.1
*sigB*	39.3 ± 3.0
*codY*	52.5 ± 1.2
*saHPF*	28.4 ± 1.8

a The percentage of live/dead bacteria of each strain was determined in triplicate with at least two biological replicates. The mean values of the percentages of live bacteria are indicated.

### Differential Invasive and Adhesive Properties of *sigB*, *codY* or *saHPF* Mutant Bacteria toward Human Lung Epithelial Cells

**Figure 3:**
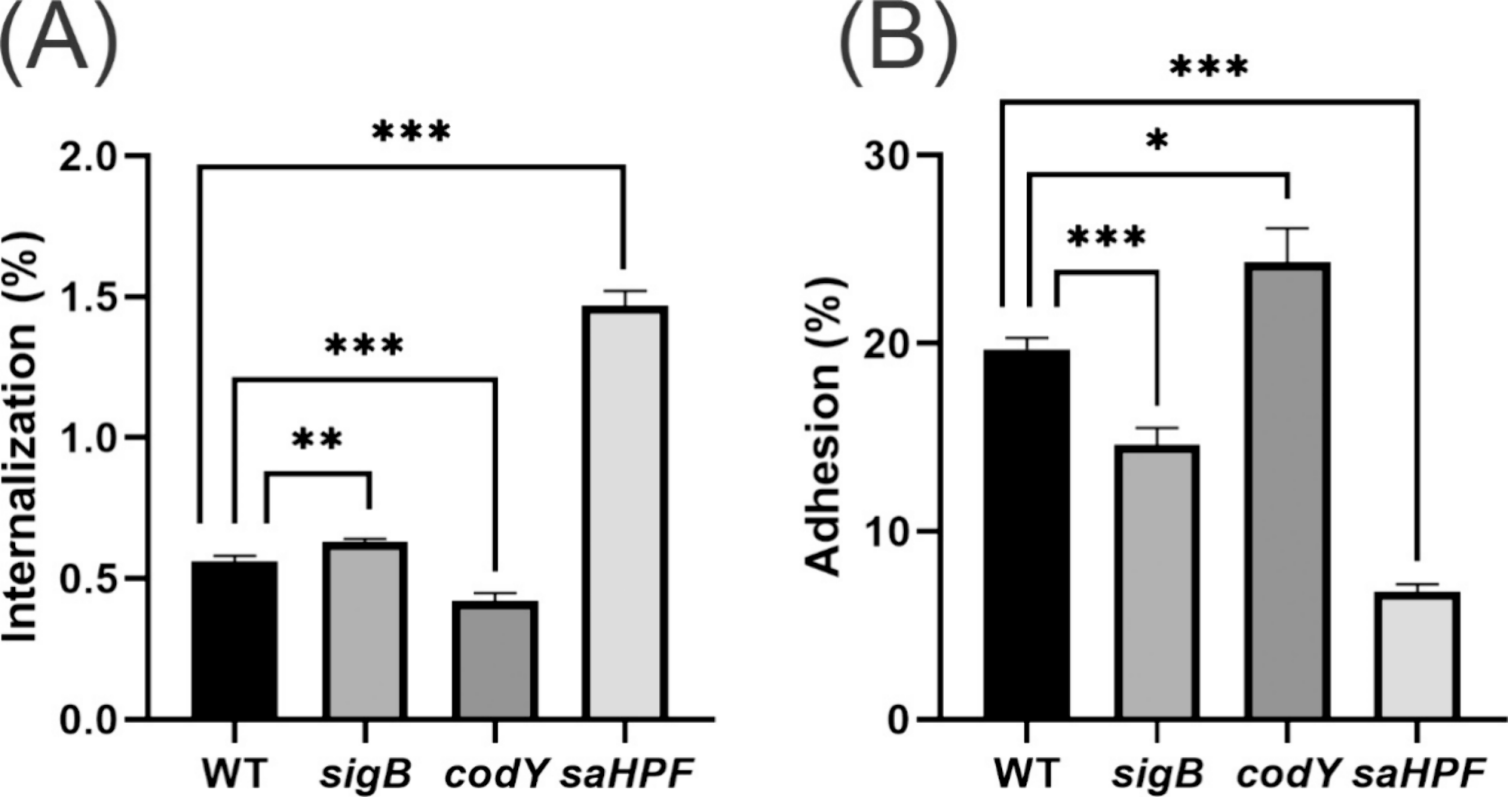
Invasive and adhesive behavior of *sigB, codY* or *saHPF* mutant bacteria toward human lung epithelial cells. The percentage of internalized (A) or adherent (B) bacteria relative to the total number of bacteria added to 16HBE14o- human lung epithelial cells at a MOI of 100 was determined for the USA300 WT strain and the respective *sigB*, *codY* or *saHPF* mutant bacteria. In this experiment, the bacteria and cells were first coincubated for 1 h in the absence of lysostaphin. Subsequently, the medium was replaced with fresh medium, and incubation was continued for 30 min in the presence of lysostaphin to measure bacterial internalization, or in the absence of lysostaphin to measure adhesion. The numbers of internalized or adherent bacteria were then determined by CFU counting on BA plates. The bar plots show the mean percentages from three individual experiments and the error bars show the standard deviation. Statistical significance was assessed by two-tailed *t* tests. A P-value <0.05 was considered significant (*, *p* < 0.05; **, *p* < 0.01; ***, *p* < 0.005).

To investigate the invasive behavior of the *sigB*, *codY* or *saHPF* mutant bacteria toward human lung epithelial cells, infection experiments were performed with 16HBE14o- human lung epithelial cells. To this end, the cells were exposed for 1 h to the bacteria at a MOI of 100 and, subsequently the bacterial internalization and adhesion were measured. Interestingly, the *sigB* mutant, and even more so the *saHPF* mutant, showed significantly higher internalization than the WT USA300 strain (Figure 3A). Conversely, the *codY* mutant showed a lower internalization compared to the WT strain. The inverse trend was observed when the adhesive behavior of the bacteria was assessed (Figure 3B). Significantly higher numbers of *codY* mutant bacteria adhered to the lung epithelial cells than was observed for the WT strain, whereas relatively lower numbers of the *sigB* and *saHPF* mutant bacteria adhered to the lung epithelial cells (Figure 3B). Altogether, these results show that the *saHPF* mutant bacteria have the highest tendency to invade the lung epithelial cells, whereas the *codY* mutant bacteria preferentially adhere to the lung epithelial cell surface.

### Impact of Mutations in *sigB*, *codY* or *saHPF* on Infection Dynamics of Lung Epithelial Cells

**Figure 4:**
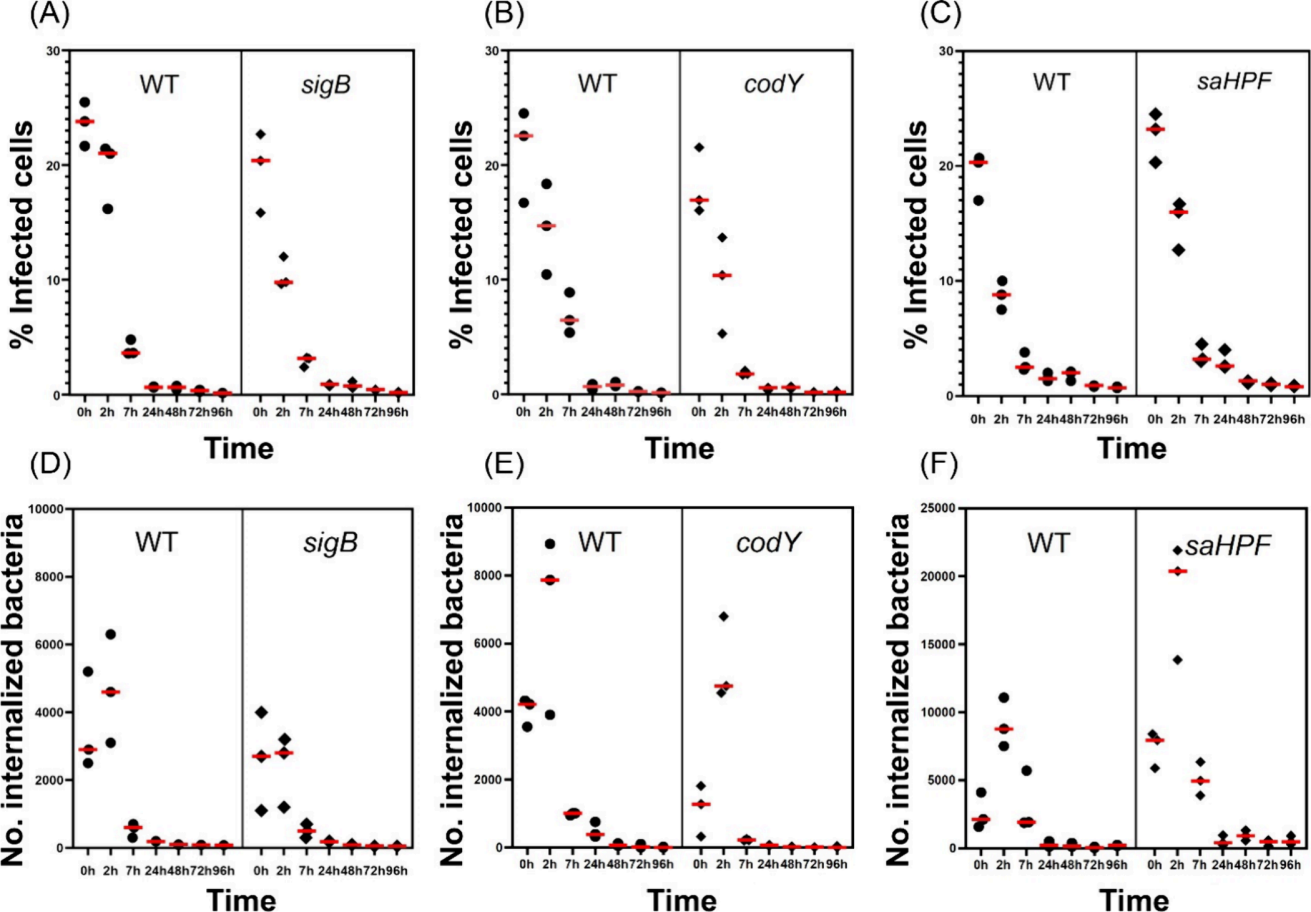
Percentage of infected lung epithelial cells and numbers of internalized *sigB, codY* or *saHPF* mutant bacteria. Confluent human lung epithelial cells were infected for 1 h with USA300 WT bacteria, or *sigB*, *codY* or *saHPF* mutant bacteria and, subsequently, the noninternalized bacteria were eliminated by 30 min incubation with lysostaphin. The end of this 30 min incubation was defined as *t* = 0. (A-C) The percentage of cells infected by the bacteria was subsequently recorded by flow cytometry over 96 h. To this end, the cells were collected by treatment with trypsin-EDTA prior flow cytometry. (D-F) The numbers of internalized WT or mutant bacteria were followed over 96 h after the addition of lysostaphin. To this end, the cells were lysed with 0.05% SDS at different time points and the released bacteria were collected and counted by flow cytometry. Please note the different scale of the *y*-axis in panel F.

To monitor the dynamics of both the internalized bacteria and the infected host cells over time, internalization experiments were performed. To ensure an optimal reproducibility of the experiments, the infection experiments with the USA300 WT strain and each of the mutant strains were performed pairwise. For technical reasons, the different pairs had to be analyzed on different days. In each infection experiment, the cells were infected for 1 h at a MOI of 100. Subsequently, lysostaphin was added to eliminate the noninternalized bacteria and the numbers of infected cells as well as the numbers of internalized bacteria were determined by flow cytometry at 2, 7, 24, 48, 72, and 96 h after the addition of lysostaphin. The percentage of infected cells is presented in Figure 4 (A-C), showing decreasing numbers from the moment of lysostaphin addition. Notably, compared to the cells infected with the WT USA300 bacteria, the percentages of infected cells declined much faster for the *sigB* or *codY* mutant bacteria (Figure 4A,B). Cells infected with WT bacteria and *sigB* or *codY* mutant bacteria remained detectable until the 48 h time point. In contrast, the percentage of cells infected with *saHPF* mutant bacteria declined slower than was observed for the WT bacteria (Figure 4C). These observations show that the *sigB* and *codY* mutant bacteria can be cleared more efficiently by lung epithelial cells compared to WT USA300, whereas the *saHPF* mutant bacteria are capable of colonizing a larger proportion of host cells for longer time periods compared to the WT. This view was validated by measuring the numbers of internalized bacteria (Figure 4, D-F). Here, a significant increase in the number of internalized bacteria was detected for all the strains at the 2 h time point compared to time 0 h, which likely reflects intracellular growth. This observed increase in intracellular bacteria was most and least pronounced for the *saHPF* and *sigB* mutant strain, respectively. Interestingly, the numbers of internalized bacteria dropped sharply at the 7 h time point for all investigated strains. Overall, the results show that the numbers of internalized bacteria measured for the *sigB* and *codY* mutant strains were lower than those of the WT bacteria up until the 7 h time point (Figure 4D,E). The WT and *codY* mutant bacteria became essentially undetectable between 24 and 48 h after lysostaphin treatment, whereas *sigB* mutant bacteria persisted until the 48 h time point. In contrast, the number of internalized *saHPF* mutant bacteria was higher than that of the WT bacteria during the entire course of the infection experiment (Figure 4F).

### Co-localization of Internalized *sigB*, *codY* or *saHPF* Bacteria with the Lysosomal Marker LAMP-1

**Figure 5:**
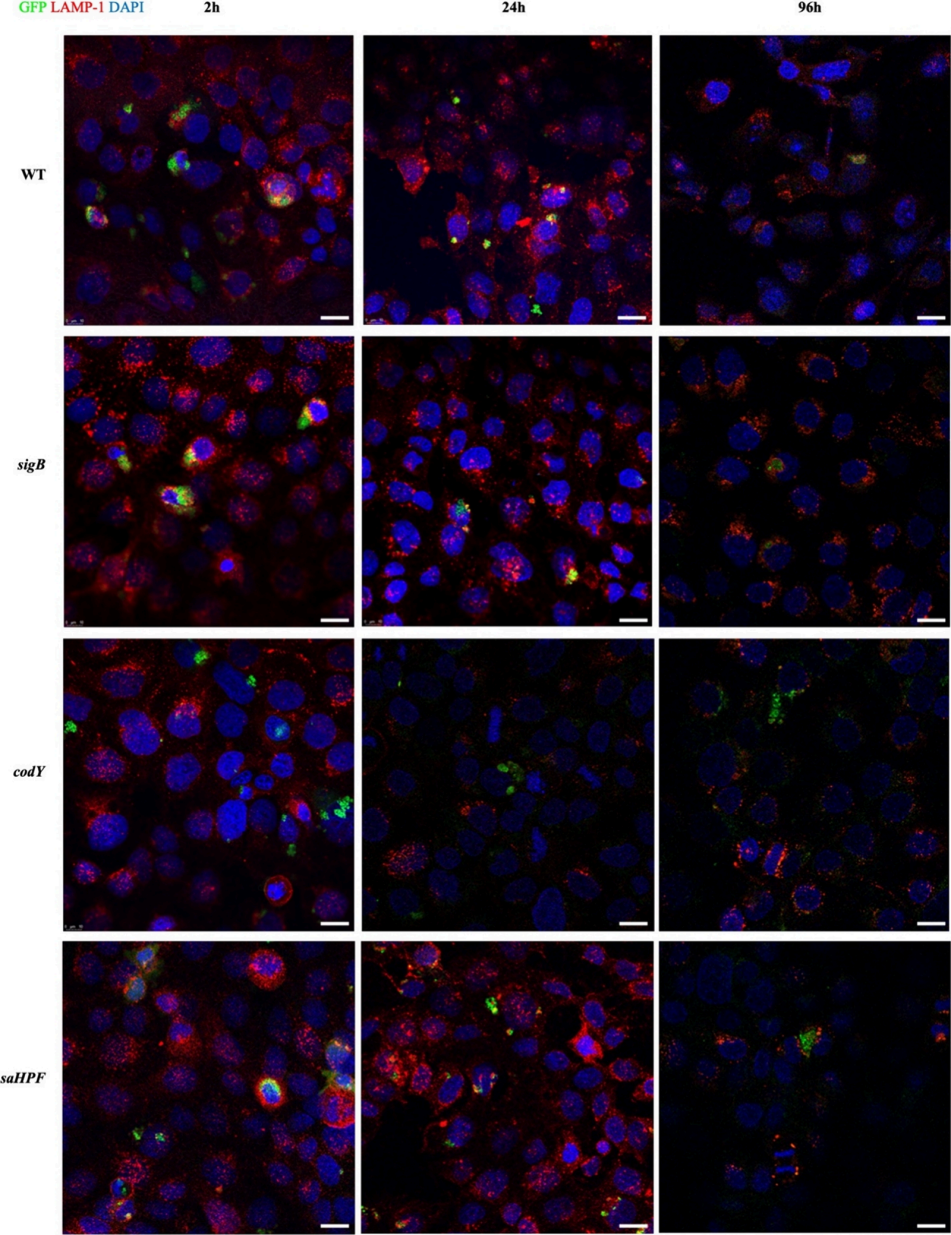
Co-localization of internalized *sigB, codY* or *saHPF* mutant bacteria with the lysosomal marker LAMP-1 in lung epithelial cells. The subcellular localization of internalized bacteria was examined by inspecting their possible colocalization with the lysosomal marker protein LAMP-1. Confluent lung epithelial cells on coverslips were challenged with the different bacteria for 1 h at an MOI of 100 and, subsequently, the extracellular bacteria were eliminated by 30 min incubation with lysostaphin. Next, at *t* = 0, the samples collected at different intervals were fixed with 4% paraformaldehyde, permeabilized, and blocked. To visualize LAMP1, the samples were incubated with the primary CD107a antibody (i.e., anti-LAMP-1) and a secondary goat antirabbit antibody labeled with Alexa 594 (red). The localization of the internalized bacteria was visualized by virtue of their GFP expression (green). Nuclei of the infected cells were stained with DAPI (blue). Confocal laser scanning fluorescence microscopy was performed with a Leica TCS SP8 microscope. Scale bars in the images indicate 20 μm.

Confocal fluorescence microscopy was applied to determine the possible colocalization of internalized bacteria with the human LAMP-1 protein, because this protein is recruited to lysosomal, phagolysosomal and vacuolar membranes. Images were recorded at 2, 24, and 96 h after the addition of lysostaphin to eliminate noninternalized bacteria (Figure 5). The results show that internalized USA300 WT bacteria colocalized with LAMP-1 from 2 until 96 h. Similarly, we observed that the internalized *sigB* or *saHPF* mutant bacteria colocalized with LAMP-1. In contrast, the internalized *codY* mutant bacteria did not colocalize with LAMP-1. Instead, the *codY* mutant bacteria were detectable in the cytoplasm of the infected cells over the entire time course of the experiment. In accordance with the flow cytometry data in Figure 4, the numbers of internalized bacteria that were detectable by microscopy declined steadily over the 96 h of the experiment. These observations show that the *codY* mutant bacteria reached a different subcellular localization in human lung epithelial cells than the WT or the *sigB* and *saHPF* mutant bacteria.

### Cytotoxicity of *sigB*, *codY* or *saHPF* Mutant Bacteria toward Lung Epithelial Cells

**Figure 6:**
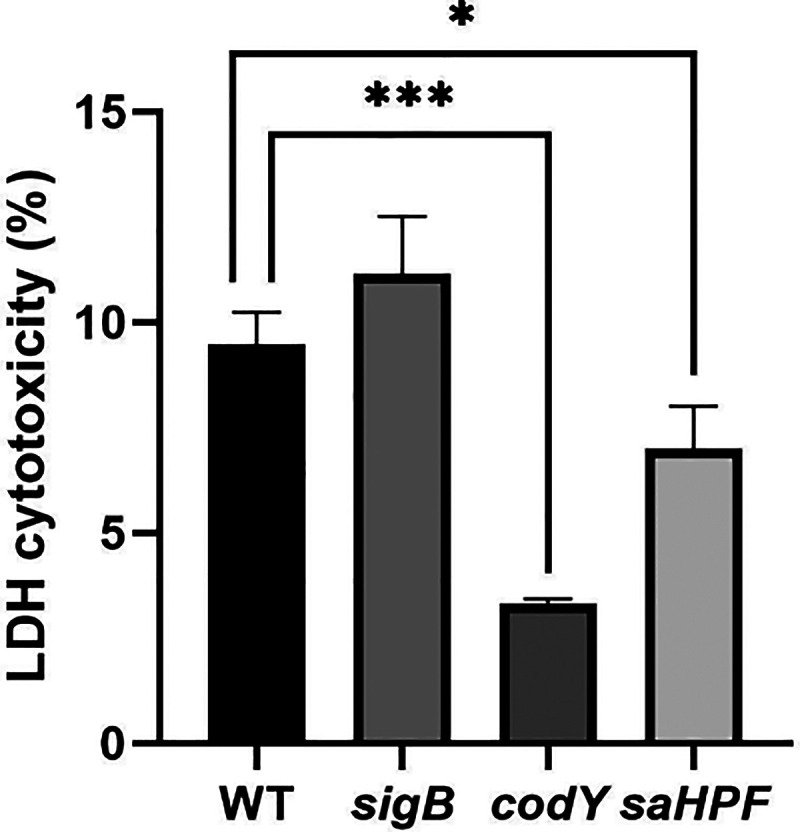
Cytotoxicity of *sigB, codY* or *saHPF* mutant bacteria toward human lung epithelial cells. Human lung epithelial cells were infected with the WT USA300 bacteria, or with *sigB*, *codY* or *saHPF* mutant bacteria for 1 h at a MOI of 100. Next the lung epithelial cell lysis was evaluated by measuring the release of LDH. The statistical significance of differences in LDH release was assessed by two-tailed *t* tests. A P-value <0.05 was considered significant. Statistically significant differences are marked (*, *p* < 0.05; ***, *p* < 0.005).

To assess the cytotoxicity of the *sigB*, *codY* or *saHPF* mutant bacteria toward human lung epithelial cells, the release of LDH into the cell culture medium after a 1 h bacterial challenge at a MOI of 100 was quantified. This revealed a significantly lower cytotoxicity of the *codY* and *saHPF* mutant bacteria compared to the WT bacteria (Figure 6). Here it is noteworthy that the *saHPF* mutant bacteria were less cytotoxic than the WT bacteria, despite their higher internalization and persistence. The cytotoxicity of the *sigB* mutant bacteria trended to be higher than that of the WT, but the difference was not significant. Altogether, the present results show a significantly decreased virulence of the *codY* mutant bacteria toward human lung epithelial cells.

### Comparative Proteome Profiling of USA300 WT and Isogenic *sigB*, *codY* or *saHPF* Mutant Bacteria

**Figure 7:**
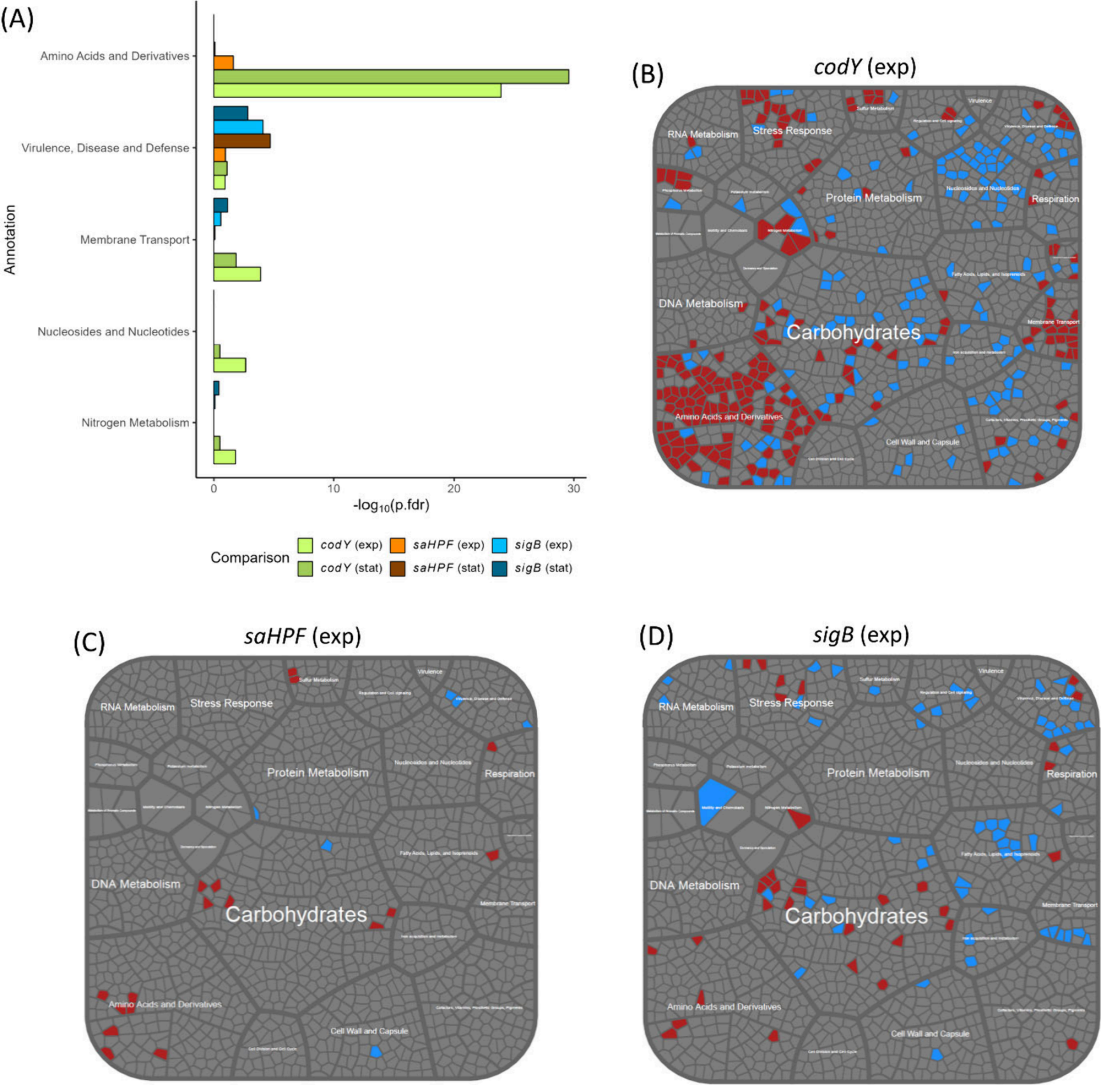
Enrichment analysis and Voronoi treemaps representing the functions of cellular proteins of the *sigB, codY* or *saHPF* mutant bacteria. (A) Enrichment analysis of functional categories according to TheSEED.^46^ Functional categories with at least five detected proteins and significant enrichment in at least one of the comparisons (mutant vs USA3000 WT in exponential or stationary growth phase) are depicted. (B, C, D). The known functional categories according to TheSEED^46^ of identified proteins are visualized as Voronoi treemaps taking into account all functional categories. The size of each functional category is proportional to the number of identified proteins belonging to the respective functional category. Red represents proteins that were detected as significantly elevated compared to the USA300 WT strain, blue represents proteins detected as significantly reduced and gray represents proteins with no significant difference in abundance. The different treemaps show the relative abundances of cellular proteins (B) from *codY* mutant bacteria in the exponential growth phase, (C) *codY* mutant bacteria in the stationary growth phase, (D) *sigB* mutant bacteria in the exponential growth phase. Voronoi treemaps are additionally shown in greater detail for exponential and stationary growth phase comparisons in Supplemental Figure S3.

To associate the observed antimicrobial resistance and infectious behavior of the *sigB*, *codY* or *saHPF* mutant bacteria with physiological changes due to these three mutations, a proteome analysis of the bacterial cells cultivated in RPMI and harvested during exponential growth and the early stationary phase was performed. The focus was placed on the cellular proteome, because our previous study on the proteome dynamics of *S. aureus* bacteria internalized by lung epithelial cells identified mostly cellular proteins controlled by SigB or CodY, and SaHPF.^14^ Moreover, in our setup for the present infection experiments, we applied in eMEM medium diluted exponentially growing *S. aureus* bacteria, which are known to secrete relatively few proteins.^47,48^ The already secreted proteins at the time point of sample preparation will therefore contribute only to a very minor extent, if any, to infection. In total 1700 *S. aureus* proteins were identified with at least two peptides (Table S2). Each mutant was compared to the USA300 WT in the respective growth phase to analyze strain-specific changes in the proteome profile. The strongest effects in terms of differential cellular protein abundance compared to the USA300 WT bacteria were observed for the *sigB* (exp: 109 proteins; stat: 143 proteins) or *codY* mutant bacteria (exp: 306 proteins; stat: 134 proteins), with relatively minor proteome changes being observed in the *saHPF* mutant bacteria (exp: 21 proteins; stat: 28 proteins) as shown in Supplemental Figure S5. This was demonstrated by a principal component analysis (PCA), where the different sample groups of *sigB* or *codY* mutant bacteria were separated from the samples of the wild-type bacteria, both in the exponential and stationary growth phases. In contrast, the samples of the *saHPF* mutant bacteria clustered with the samples of the WT bacteria (Supplemental Figure S2). However, closer investigation of the differentially abundant proteins revealed that the proteome changes observed in the *saHPF* mutant bacteria showed a similar tendency as observed in the *sigB* mutant bacteria and, to lesser extent, in the *codY* mutant bacteria (Supplemental Figures S3 and S4). Overviews of the biological functions of the differentially abundant proteins of the *codY, sigB* or *saHPF* mutant bacteria compared to the USA300 WT are given in Voronoi treemaps based on the functional “TheSEED”^46^ annotation of the *S. aureus* genes (Figure 7, Supplemental Table S2). These Voronoi treemaps assign functionally related proteins to the same cluster and partition them into weighted polygons with an area proportional to the relative weight of the respective functional category.

In the *codY* mutant bacteria, proteins present at elevated levels perform mainly roles in metabolic processes related to ‘Amino Acids and Derivatives’ (fdr-adjusted p-value of Fisher’s Exact enrichment test of significantly different protein abundances using the functional categories based on the first “TheSEED” category (Supplemental Table S3): 4.3e-24 (exp); 7.3e-31 (stat)), “Membrane Transport” (1.5e-4 (exp); 1.2e-2 (stat)), and “Nitrogen Metabolism” (3.8e-3 (exp)) (Figures 7A,B, Supplemental Figure S3). In contrast to the clear metabolic effects of CodY-deficiency, the proteins detected at elevated levels in the *sigB* mutant bacteria compared to the USA300 WT belong to a broad set of functional categories and the proteins detected at reduced levels in the mutant are involved in processes related to ‘Virulence, Disease and Defense’ (1.3e-4 (exp); 3.6e-4 (stat)) and ‘Fatty Acids, Lipids, and Isoprenoids’ (Figure 7A,D, Supplemental Figure S3).

For the *saHPF* mutant bacteria, it was observed that proteins present at elevated levels in the exponential growth phase are mainly involved in processes related to ‘Amino Acids and Derivatives’ (2.0e-2 (exp)), whereas the proteins present at reduced levels in the exponential and stationary growth phase serve in processes related to ‘Virulence, Disease and Defense’ (3.5e-5 (stat)), and “Cell Wall and Capsule” and (Figure 7A,C, Supplemental Figure S3).

Altogether, these observations are in line with the results from previous studies on the roles of CodY and SigB in the metabolism and virulence of *S. aureus*,^14,49^ and they are consistent with our presently observed differences in antimicrobial resistance and virulence. A heatmap showing all *S. aureus* proteins detected at elevated or reduced levels in the *sigB*, *codY* or *saHPF* mutant bacteria compared to the USA300 WT strain is shown in Supplemental Figure S4.

### The *sigB*, *codY* or *saHPF* Mutant Bacteria Show Altered Virulence Factor Profiles

To associate the proteomics data with the observed features of the *sigB*, *codY* or *saHPF* mutant bacteria, we evaluated which of the proteins that were detected at significantly different levels compared to the USA300 WT bacteria were related to antimicrobial resistance or host cell adhesion and cytotoxicity. Based on the functional TheSEED annotation, 15 of the identified proteins are involved in antimicrobial resistance, 19 in adhesion to host cells and 5 in cytotoxicity. A summary of the results of differently abundant proteins per strain and its growth phase is presented in Supplemental Table S4, and detailed information on the respective protein functions is provided in Supplemental Table S2.

**Figure 8:**
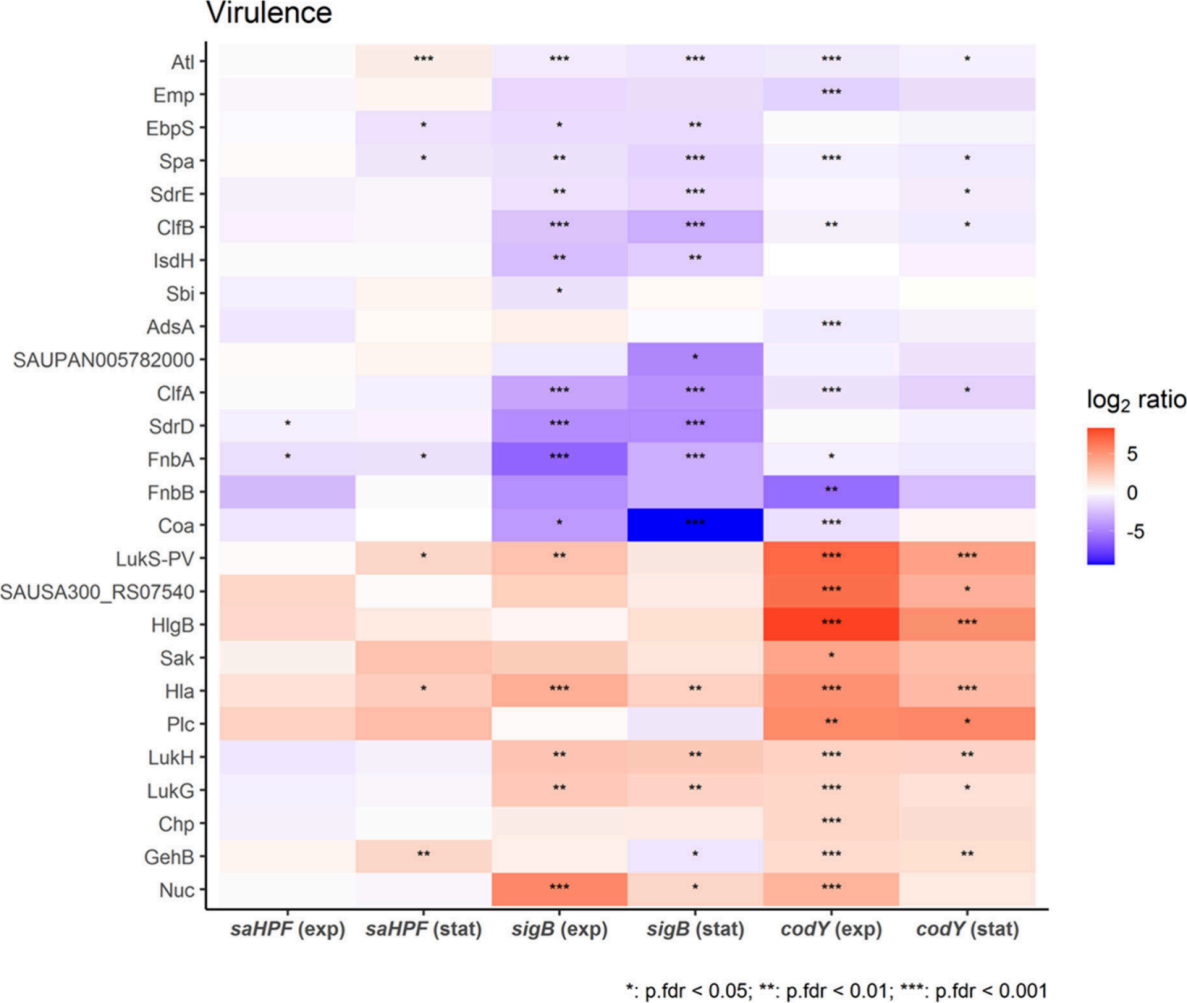
Heatmap showing the 26 known *S. aureus* virulence factors present at elevated or reduced levels in the *sigB, codY* or *saHPF* mutant bacteria compared to the USA300 WT strain. The relative abundance of the included virulence factors in the different mutant bacteria compared to the WT bacteria was determined based on normalized median peptide intensity and is indicated by color-coded bars. The ROPECA statistical significance is indicated by stars. Of note, the included virulence factors were annotated based on TheSEED categories “resistance”, “adhesion”, “cytolysin” or “virulence” as described by Mäder et al. (2016), but the CopA, SAUPAN005717000, PurF, GyrA, HslO, GraR, GraS, and EmrB proteins were omitted because they are not directly involved in virulence.

A total number of 67 known *S. aureus* virulence factors was identified in this proteome profiling experiment, including 26 proteins displaying significantly different levels in the *sigB*, *codY* or *saHPF* mutant bacteria compared to the *S. aureus* USA300 WT bacteria (Figure 8). Deficiency of CodY, SigB and SaHPF resulted in general in two clusters of elevated and reduced virulence factors. In *codY* mutant bacteria, the elevated cluster and especially the HlgB (γ-hemolysin subunit), LukS-PV (Panton-Valentine leukocidin subunit S), LukF-PV (Panton-Valentine leukocidin subunit F; SAUSA300_RS07540) and Plc (1-phosphatidylinositol phosphodiesterase) proteins were more strongly elevated. In contrast, the reduced cluster and especially the Coa (coagulase), FnbA (fibronectin-binding protein A), SdrD (serine-aspartate repeat-containing protein D), SAUPAN005782000 (putative autolysin), ClfA (clumping factor A), IsdH (iron-regulated surface determinant protein H), and ClfB (clumping factor B) proteins were more strongly reduced in the *sigB* mutant bacteria. Furthermore, compared to the USA300 WT bacteria, the fold changes in protein levels were generally less extreme in the *saHPF* mutant bacteria. However, unlike the *sigB* and *codY* mutant bacteria, the *saHPF* mutant bacteria presented elevated levels of Atl (autolysin) and slightly reduced levels of LukGH (leukocidin).

Altogether, these mutant-specific changes in the levels of virulence factors provide an explanation for the differential behavior of the *codY*, *sigB* or *saHPF* mutant bacteria in our different virulence assays.

## Discussion

In a previous proteomics investigation on the adaptive behavior of *S. aureus* internalized by human lung epithelial cells, we observed that especially SigB- and CodY-regulated proteins showed an altered abundance over time.^14^ This suggested that the respective regulons would have important roles in the adaptation to the intracellular environment and subsequent persistence inside the epithelial cells. In addition, the abundance of SaHPF increased dramatically immediately upon internalization, suggesting a role for this protein in intracellular growth and survival. Therefore, we now explored the roles of SaHPF, SigB and CodY in the infectious behavior and intracellular survival of *S. aureus* upon internalization by human lung epithelial cells, using bacteria with transposon insertions in the respective genes. Indeed, our present results reveal that the different mutations have a significant impact on the bacterial ability to adhere to and invade human lung epithelial cells. In fact, the three tested mutations do not only impact host cell adhesion and invasion, but they also influence the bacterial growth, division and resistance to antibiotics. In particular, the *codY* mutant showed a behavior that is reminiscent of SCVs, with slowed-down *in vitro* growth and increased antibiotic resistance. This is, in fact, the type of behavior that we anticipated to observe, based on our previous infection studies, where the internalized bacteria displayed metabolic adaptations that were reminiscent of those of SCVs. The main difference here was that over the relatively short period of measurement (96 h) in our previous study no real SCVs emerged among the internalized bacteria, implying that the phenotype of the internalized bacteria was still reversible. Clearly, this differs from the present experimental setup, where mutant bacteria already exhibited the respective stable phenotype at the start of the infection experiment and had fewer opportunities to adapt to the intracellular environment compared to wild-type bacteria. This is clearly evidenced not only by growth differences *in vitro*, but also by other features as exemplified by the *sigB* mutant bacteria, which showed a reduced yellow pigmentation by the antioxidant staphyloxanthin and increased hemolytic behavior already at the start of the infection experiment. These features of *sigB* mutant *S. aureus* were, in fact, also previously reported.^50,51^

Our present lung epithelial cell infection experiments focus particular attention on the CodY-perceived metabolic state of the bacteria with respect to host cell adherence, invasion, intracellular persistence and cytotoxicity, because all these aspects of infection were influenced by a transposon insertion in *codY*. The importance of CodY in probing the bacterial metabolic state is most clearly reflected by the functional enrichment analysis of the proteome data, which revealed strong effects of the *codY* mutation on the levels of staphylococcal proteins involved in amino acid metabolism, nucleoside and nucleotide metabolism and nitrogen metabolism. Altogether, we identified 93 known CodY-regulated proteins. In the *codY* mutant 63 of these proteins were upregulated and 2 downregulated during exponential phase, whereas 46 were upregulated and 1 was downregulated during stationary phase (Supplemental Figure S5). With respect to the most strongly upregulated proteins, there is a noteworthy overlap with the CodY-regulated proteins for which we previously documented upregulation during intracellular staphylococcal persistence in human lung epithelial cells, especially metabolic enzymes like Asd, ButA, DapABD, Hom, MetE, PycA, SerA, ThrC and most proteins encoded by the *ilv*-*leu* operon which are required for BCAA synthesis.^14^ On the other hand, unlike in our previous study,^14^ we observed in the *codY* mutant significant downregulation of the IlvA1 protein and no regulation of ArgG, whereas AcsA was not detected. The latter differences could perhaps be relevant with respect to intracellular persistence as, in our present study, the *codY* mutant bacteria were more rapidly cleared by the lung epithelial cells than the wild-type bacteria. It remains to be shown whether this difference and other less eminent differences in the levels of CodY-regulated proteins as observed in our previous study and the present one are due to bacterial strain-specific differences and/or differences in the conditions to which the bacteria were exposed, especially intracellular growth of wild-type bacteria versus growth of *codY* mutant bacteria in RPMI medium. Furthermore, in accordance with our present phenotypic analysis, previous studies have demonstrated that mutations in genes that are regulated by CodY relate to the formation of SCVs in *S. aureus*.^8,52^ Also, it is known that the Agr and SaeRS regulatory systems have a role in SCV formation by modulating the expression of important virulence factors of *S. aureus*.^13,53^ Since CodY is a known regulator of Agr and SaeRS,^13,54^ it is conceivable that the *codY* mutant displayed a SCV phenotype through altered regulation of Agr and SaeR. This is consistent with the results of our present proteomics studies, showing that the abundance of several virulence factors that are regulated by CodY or SaeR was changed in the *codY* mutant. For example, we observed that the abundance of the BrnQ1 and BrnQ2 proteins for putative BCAA transporters increased in the *codY* mutant (Supplemental Table S2). In a murine infection model, it was previously demonstrated that the virulence of *brnQ2* mutant bacteria was significantly increased compared to that of the USA300 strain.^55^ Conversely, the observed increase in the level of BrnQ2 could explain the lowered virulence phenotype of the *codY* mutant bacteria. As mentioned in another previous study, the GltB protein is involved in the formation of late exponential phase *S. aureus* persisters by interaction with PurN and it also regulates *S. aureus* virulence by activating the SaeRS two-component system.^56^ However, despite the presently observed increase in the abundance of GltB in the *codY* mutant bacteria (Supplemental Table S2) and despite their SCV phenotype, the *codY* mutant bacteria did not display a persistent phenotype in human lung epithelial cells. In addition, the abundance of the SodM was significantly increased in *codY* mutant bacteria. A prior study reported the importance of SodM for *S. aureus* virulence and persistence within the airways of patients with cystic fibrosis.^57^ Yet, the elevated level of SodM was apparently not sufficient to promote intracellular persistence of the *codY* mutant bacteria in our experimental setup. On the other hand, we also observed increased levels of the bicomponent leukocidin LukGH in the *codY* mutant bacteria. LukGH is an important virulence factor of *S. aureus*, which lyses human phagocytic cells and contributes to immune evasion.^58^ Nonetheless, the cytotoxicity of the *codY* mutant bacteria toward lung epithelial cells was reduced in the current study. In this respect, it is important to bear in mind that cytotoxicity depends on many different factors, and that LukGH is only one of many cytotoxins produced by *S. aureus*, including α-hemolysin (Hla) and Panton-Valentine leukocidin (PVL). These factors collectively contribute to the overall cytotoxic profile of the bacterial strain. Furthermore, it has been reported that *S. aureus* SCVs show increased resistance to many antibiotics,^59,60^ which explains why SCVs are more difficult to eradicate by antimicrobial therapy and give rise to chronic infections. Consistent with this notion, the *codY* mutant strain was found to be more resistant to benzylpenicillin, vancomycin and nitrofurantoin than the wild-type strain in the current study. Our proteomics analyses now show that several antimicrobial resistance-associated proteins were upregulated in the *codY* mutant bacteria, but not in the *sigB* and *saHPF* mutants. For instance, this concerned the Alr2 protein (Supplemental Table S2), which is involved in cell wall biosynthesis and vancomycin resistance of *S. aureus*.^61^

Our present study shows that also SigB plays an important role in lung epithelial cell infection by *S. aureus*, since host cell adherence, invasion and intracellular persistence were all influenced by a transposon insertion in the *sigB* gene. In particular, the *sigB* mutant bacteria were more rapidly cleared from the host cells than the wild-type bacteria. This implies that SigB-perceived stresses need to be adequately addressed by the bacteria during infection to warrant intracellular survival and persistence. This is possibly achieved through SigB-dependent production of virulence factors as indicated by the functional enrichment analysis of our proteome data, which revealed the strongest effects of the *sigB* mutation on proteins involved in virulence. Altogether, we identified 117 SigB-regulated proteins of which 49 were significantly less abundant in the *sigB* mutant compared to the wild-type in the exponential phase and 50 in the stationary phase (Supplemental Figure S5). In our previous proteomics study on the adaptation of *S. aureus* to the intracellular conditions in lung epithelial cells, a role for SigB in reaching the dormancy state was suggested by changes in the abundance of 58 proteins that are known to be regulated by SigB.^14^ However, this did not only involve downregulated proteins but also upregulated proteins, whereas we observed only reduced levels of SigB-regulated proteins in the *sigB* mutant bacteria in our present study. It thus seems that SigB is needed for correct bacterial decision taking upon host cell invasion through appropriate expression of at least a subset of the SigB-regulated proteins. At present, we do not know the precise nature of the stresses perceived by SigB during the different stages of lung epithelial cell infection, but nutrient stress could be one of them.^14^ In particular, we have previously shown that the RPMI medium used in our present study is an iron-restricted medium that closely resembles the conditions in human plasma.^37^ Thus, some of the effects observed in our study could relate to the limited availability of iron, which is a typical condition that invasive pathogens must face in the human body. Furthermore, in contrast to the *codY* mutant, the *sigB* mutant, and especially the *saHPF* mutant, showed decreased resistance to benzylpenicillin compared to the wild-type strain, which implies a potential role of SaHPF in modulating antibiotic resistance. This result may be explained by the fact that the known methicillin-resistance associated proteins MecA and UgpQ^62^ were found to be downregulated in the *sigB* and *saHPF* mutant bacteria in our present study. This observation is also in keeping with results from previous studies, which indicated that *sigB* plays a role in the resistance of *S. aureus* to cell wall-active antibiotics by regulating the expression of some proteins, like the methionine sulfoxide reductase MsrA and the major chaperone GroEL.^63,64^ However, while there was a trend toward upregulation of MsrA1, MsrA2 and GroEL in the *sigB* mutant, both in the exponential and stationary growth phases, these changes were not statistically significant. Like SigB, SaHPF is crucial for the survival of *S. aureus* in harsh environments and, as shown in our present study, this extends to antibiotic exposure. It remains, however, to be investigated why exactly the *saHPF* mutant is more sensitive to benzylpenicillin and nitrofurantoin. For instance, this could relate to the role of SaHPF in the modulation of ribosomal activity which, in turn, could lead to altered expression of penicillin-binding proteins. Furthermore, the influence of the *saHPF* mutation on nitrofurantoin resistance could be explained by the fact that the reduction of nitrofurantoin by nitroreductases of *S. aureus* will yield intermediate products that target ribosomal proteins, thereby further reducing protein synthesis.^65^

Importantly, our present study highlights a differential adhesion and internalization by human lung epithelial cells of the *sigB*, *saHPF* or *codY* mutant bacteria. Interestingly, the *codY* mutant bacteria displayed an increased tendency to adhere to the cells, whereas the opposite effect was observed for the *sigB* and *saHPF* mutant bacteria. This may be explained by the observed downregulation of the Atl and FnbA proteins in the *sigB* and *saHPF* mutant bacteria, which are important for biofilm formation and colonization of host cells.^66,67^ Conversely, the internalization of *saHPF* mutants was strongly enhanced compared to the WT USA300 bacteria and, to lesser extent, this was also true for *sigB* mutant bacteria. Notably, reduced numbers of internalized *codY* mutant bacteria were observed. Together these observations imply that the *codY* mutant bacteria have a lower tendency to invade lung epithelial cells, whereas the *saHPF* and *sigB* mutant bacteria have an increased propensity for invasive behavior. It was previously shown that expression of the *clfA* and *fnbA* genes, encoding important adhesins of *S. aureus*, is regulated by SigB.^68^ Accordingly, our present proteomics analyses show that the FnbA protein level is reduced in the *sigB* or *saHPF* mutant bacteria. Furthermore, ClfA was found to be downregulated in the *sigB* mutant bacteria. Together, these observations imply that a reduced abundance of FnbA and ClfA could limit the retention of the *sigB* and *saHPF* mutant bacteria at the cell surface and, consequently, favor an increased invasive behavior. On the other hand, a strong relationship between SCV formation and upregulated expression of adhesion genes in *S. aureus* has been reported previously.^69,70^ This could explain the enhanced adhesion of the *codY* mutant bacteria,^71,72^ but it should be noted that we did not observe an upregulation of known adhesins in the *codY* mutant bacteria in our proteome analyses. The latter may relate to the specific conditions in our experimental setup for the proteome analyses or, perhaps, the expression of alternative, yet unidentified adhesins in the *S. aureus* USA300 *codY* mutant.

The observed differences in the bacterial cytotoxicity toward host cells, as assessed by LDH release, may reflect both the cytotoxicity of the bacteria prior internalization and post internalization. Notably, the *codY* mutant bacteria showed the lowest cytotoxicity toward the investigated lung epithelial cells. The finding that the *codY* mutant bacteria had a higher tendency for adhesion than internalization, could suggest that the LDH measurements mostly reflect cytotoxicity upon internalization. However, the SCV phenotype of the *codY* mutant bacteria implies that they are also less virulent. Furthermore, this observation also accords with our earlier observations, which showed that *S. aureus* bacteria with a relatively lower tendency to internalize in host cells displayed a higher propensity for persistence within their host cells. This could relate to a dosage effect where lowered numbers of internalized bacteria gave rise to a lowered dosage of intracellular toxin production.^32^ In our present study, the *sigB* mutant bacteria displayed the highest cytotoxicity. In accordance with the present results, a previous study demonstrated that spent growth medium of a *sigB* mutant caused the highest level of cell death.^11^ The latter study also showed that the *sigB* mutation precluded SCV formation, leading to enhanced clearance of the mutant bacteria from infected cells, which is consistent with our present observations. In contrast to the *sigB* mutant bacteria, the *saHPF* mutant bacteria presented a lowered LDH cytotoxicity compared to the WT strain. This matches well with the somewhat increased intracellular persistence of these bacteria in the human lung epithelial cells. Here it is noteworthy that a previous study reported that a lack of *saHPF* expression in the *S. aureus* strain SH1000 did not affect the bacterial survival and persistence in macrophages,^73^ implying that the cytotoxicity of the bacteria was not enhanced. However, since the previous study addressed a different *S. aureus* strain and different host cells, the previous and present results cannot be compared directly.

To track the dynamic interplay between *S. aureus* USA300 and human lung epithelial cells, the numbers of infected cells and internalized bacteria were followed over 96 h p.i. by flow cytometry. There was a sharp drop of the internalized bacterial numbers at 7 h p.i. for all the strains. This could suggest that the invading bacteria trigger the defensive responses in the host cells. On the other hand, the numbers of infected cells also decreased at the 7 h time point, which shows that the internalized bacteria caused host cell lysis. In our previous study with the *S. aureus* strain HG001,^14^ we observed two subpopulations of internalized bacteria in lung epithelial cells. One subgroup started to replicate upon host cell invasion and subsequently caused cell lysis, whereas the other subgroup displayed a very low growth rate and persisted within host cells over the entire 96 h time course of the experiment. In contrast, in the present study, the numbers of internalized bacteria and the infected cells decreased over time, and there were almost no internalized bacteria and infected cells detectable at 96 h p.i. This difference could be due to the different *S. aureus* strains that were used in the two studies. One important difference between the USA300 strain used in the present study and the previously used HG001 strain is that the USA300 strain produces PVL, whereas the HG001 does not possess the genes for this potent toxin. Since we have previously shown that the 16HBE14o- cells express at least the PVL receptor CD88,^31,32^ it is conceivable that PVL contributes to the lower intracellular survival of the USA300 strain compared to the HG001 strain. In addition, PVL may contribute to the observed host cell killing prior internalization, which will also set a limit to the numbers of intracellular bacteria. However, the USA300 strain is also notorious for producing higher amounts of phenol-soluble modulins (PSMs) compared to other *S. aureus* strains,^74^ so this could also contribute to the higher cytotoxicity of this strain. Unfortunately, the PSMs remained undetected in our present proteome analyses.

Confocal fluorescence microscopy was performed to study the intracellular distribution of *S. aureus*. It was reported in previous studies that replicating internalized *S. aureus* bacteria colocalize with the LAMP-1 positive lysosomes, while the persistent subpopulation of *S. aureus* is mostly found in the cytosol of the host.^14,32^ Consistent with this prior observation, in the present study we found that the WT bacteria as well as the *sigB* or *saHPF* mutant bacteria colocalized with LAMP-1 positive lysosomes. In contrast, the *codY* mutant bacteria were found in the cytosol of the host cells, which is in line with their SCV-like behavior. Nonetheless, the *codY* mutant bacteria were rapidly cleared by the lung epithelial cells upon internalization, which is in agreement with the notion that not all *S. aureus* bacteria with an SCV phenotype will become persisters.

## Conclusions

In our present study we have examined the phenotypic characteristics of *S. aureus* USA300 *codY*, *sigB* or *saHPF* mutant bacteria and explored their behavior upon infection of human lung epithelial cells. The results show that CodY, SigB and saHPF contribute differentially to the subsequent steps in the infection process, including host cell adhesion and invasion, intracellular survival and cytotoxicity. In a previous study, we observed major changes in CodY- and SigB-regulated proteins upon prolonged intracellular bacterial persistence inside human lung epithelial cells.^14^ Consistent with this previous observation, we now report that *sigB* or *codY* mutant bacteria experience a faster intracellular clearance compared to the parental strain, which underscores the importance of these regulators for intracellular persistence. Furthermore, we show that *codY* mutant USA300 bacteria display an SCV-like phenotype. Intriguingly, while we previously showed an increase of CodY-regulated metabolic enzymes over time in the internalized bacteria,^14^ the total absence of this repressor did not lead to improved host cell invasion or intracellular survival but, instead it had the opposite effect. This implies that CodY-mediated gene regulation needs to be fine-tuned in order to support intracellular bacterial persistence. Furthermore, our present study is the first to investigate the roles of SaHPF in the infection of human lung epithelial cells, revealing parallel effects of SaHPF or SigB deficiency in host cell adhesion and invasion. Lastly, our study shows that CodY and SigB are key players in the cytotoxicity of *S. aureus* USA300 toward lung epithelial cells, which is one of the prime features of this pathogen. Our study thus focuses attention on the CodY-perceived metabolic state of the bacteria and the SigB-perceived stress in the bacterial decision taking with respect to host cell adherence, invasion, intracellular persistence and cytotoxicity. Conversely, this implies important roles for the nutritional status and bacterial stress-inducing conditions in the host for the onset and subsequent course of invasive or chronic staphylococcal disease. Future studies should therefore focus on defining more precisely the nutritional status of intracellular *S. aureus* bacteria and the exact stresses that they are exposed to at different subcellular locations. In addition, it will be important to narrow down the roles of particular SigB- or CodY-influenced regulons, like Agr and SaeRS, or individual proteins in the different stages of lung epithelial cell infection.

## Data Availability

The data sets supporting the conclusions of this article are included within the article and its additional files. The mass spectrometry proteomics data have been deposited to the ProteomeXchange Consortium via the PRIDE^44^ partner repository with the data set identifier PXD044962. Materials will be made available upon reasonable request.

## Supporting Information Available

Supplemental Figure S1. Growth of *S. aureus* USA300 WT and *sigB*, *codY* or *saHPF* mutant USA300 bacteria in RPMI medium at 37 °C as monitored by OD_600_ readings. Supplemental Figure S2. Principal-component analysis (PCA) of all identified cellular proteins of USA300 WT and *codY*, *sigB* or *saHPF* mutant bacteria. Supplemental Figure S3. Voronoi treemaps representing the functions and relative abundance of cellular proteins of the *sigB*, *codY* or *saHPF* mutant bacteria. Supplemental Figure S4. Heatmap showing the relative abundance of cellular proteins of which the levels were significantly changed in at least one of the *saHPF*, *sigB*, or *codY* mutant bacterial strains compared to the USA300 WT bacteria in the exponential (exp) or stationary (stat) growth phases. Supplemental Figure S5. Volcano plots and Fisher’s exact tests showing significantly different protein abundances in the *codY*, *sigB*, or *saHPF* mutant bacteria and the USA300 WT bacteria. (PDF)
Supplemental Table S1. (A) Parameters for reversed phase liquid chromatography (RPLC), (B) Parameters for mass spectrometry analysis in data-independent mode, (C) Spectronaut parameters used for analysis of mass spectrometry data, and (D) R packages used for data analysis and visualization. Supplemental Table S4. Summary and list of functional protein annotations in virulence, antimicrobial resistance, adhesion to host cells and cytotoxicity of the *codY, sigB* or *saHPF* mutant bacteria, relative to USA300 WT bacteria. (PDF)
Supplemental Table S2. Proteomics data and functional annotation of identified proteins. (XLSX)
Supplemental Table S3. Functional annotation enrichment tests. (XLSX)

## Abbreviations

*S. aureus*: *Staphylococcus aureus*
MRSA: methicillin-resistant *S. aureus*
SCVs: small colony variants
BA: blood agar
CA: community-acquired
RPMI: roswell park memorial institute
CFUs: colony-forming units
CLSI: clinical and laboratory standards institute
MIC: minimal inhibitory concentrations
MH: mueller hinton
eMEM: minimal essential medium
FCS: fetal calf serum
MOI: multiplicity of infection
SDS: sodium dodecyl sulfate
LAMP-1: lysosomal-associated membrane protein1
RT: room temperature
PCA: principal component analysis
