# Supplementary material for: Functional and Proteomic Dissection of the Contributions of CodY, SigB and the Hibernation Promoting Factor HPF to Interactions of Staphylococcus aureus USA300 with Human Lung Epithelial Cells
Source: J Proteome Res. 2024 Sep 20;23(10):4742–60. doi: 10.1021/acs.jproteome.4c00724 (PMC11459534; doi:10.1021/acs.jproteome.4c00724)
Supplement: Supplementary file 1 — pr4c00724_si_001.pdf [file pr4c00724_si_001.pdf]

Supplemental Figures

Functional and proteomic dissection of the contributions of CodY, SigB and the hibernation promoting factor HPF to interactions of *Staphylococcus aureus* USA300 with human lung epithelial cells

Xiaofang Li[1], Larissa M. Busch[2], Sjouke Piersma[1], Min Wang[1], Lei Liu[1], Manuela Gesell Salazar[2], Kristin Surmann[2], Ulrike Mäder[2], Uwe Völker[2], Girbe Buist[1#] and Jan Maarten van Dijl[1#*],

[1]Department of Medical Microbiology and Infection Prevention, University of Groningen, University Medical Center Groningen, Hanzeplein 1, 9700 RB Groningen, the Netherlands

[2]Interfaculty Institute for Genetics and Functional Genomics, Department Functional Genomics, University Medicine Greifswald, D-17475 Greifswald, Germany

[*]**Corresponding author**: Jan Maarten van Dijl, University of Groningen, University Medical Center Groningen, Hanzeplein 1, 9700 RB Groningen, the Netherlands, tel. +31-50-3615187, e-mail: j.m.van.dijl01@umcg.nl

[#]Equal contributions

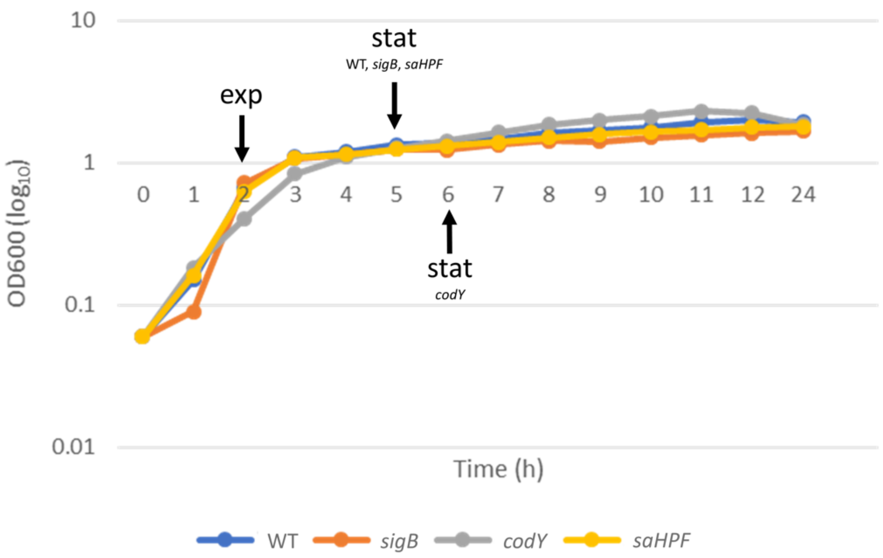

Figure S1. Growth of *S. aureus* USA300 WT and *sigB*, *codY* or *saHPF* mutant USA300 bacteria in RPMI medium at 37℃ as monitored by OD_{600} readings. The time points of sampling for proteome analyses are indicated.

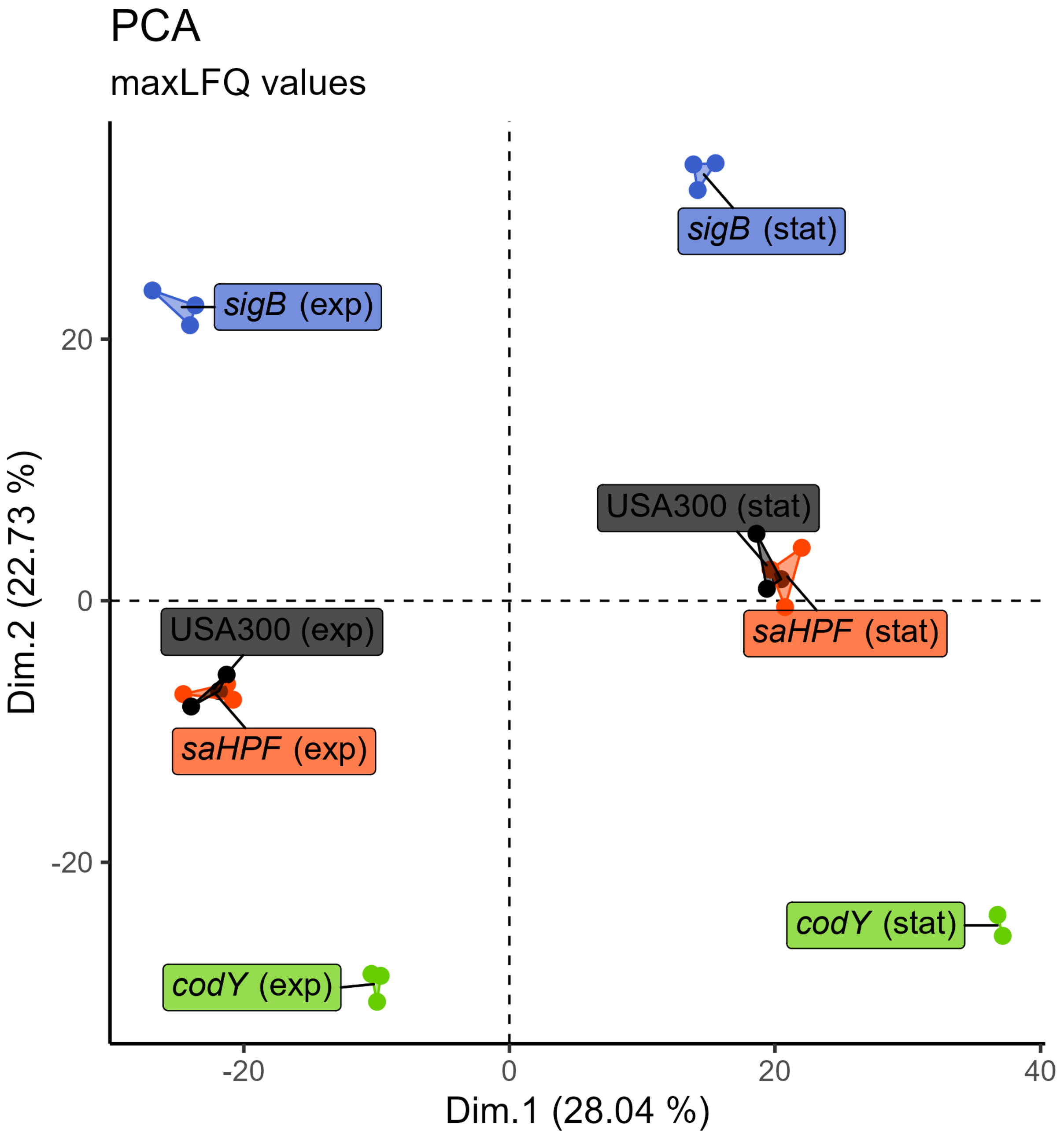

Figure S2. Principal component analysis (PCA) of all identified cellular proteins of USA300 WT and *codY*, *sigB* or *saHPF* mutant bacteria. The data obtained for the biological replicates of each condition are marked with the same color.

Figure S3

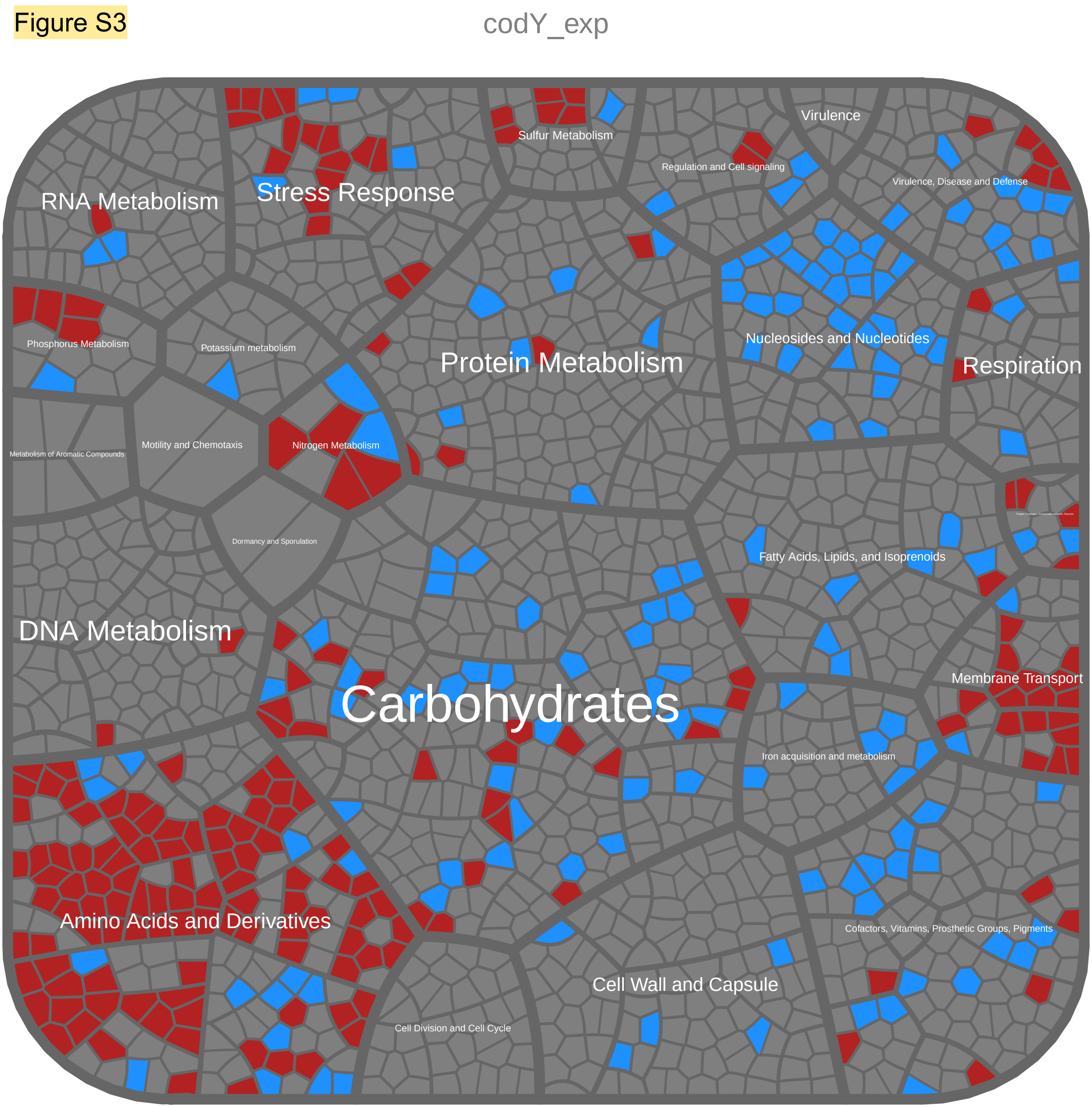
codY_exp
RNA Metabolism
Stress Response
Sulfur Metabolism
Virulence
Regulation and Cell signaling
Virulence, Disease and Defense
Phosphorus Metabolism
Potassium metabolism
Protein Metabolism
Nucleosides and Nucleotides
Respiration
Metabolism of Aromatic Compounds
Motility and Chemotaxis
Nitrogen Metabolism
Dormancy and Sporulation
Fatty Acids, Lipids, and Isoprenoids
DNA Metabolism
Carbohydrates
Membrane Transport
Iron acquisition and metabolism
Amino Acids and Derivatives
Cofactors, Vitamins, Prosthetic Groups, Pigments
Cell Wall and Capsule
Cell Division and Cell Cycle

codY_stat

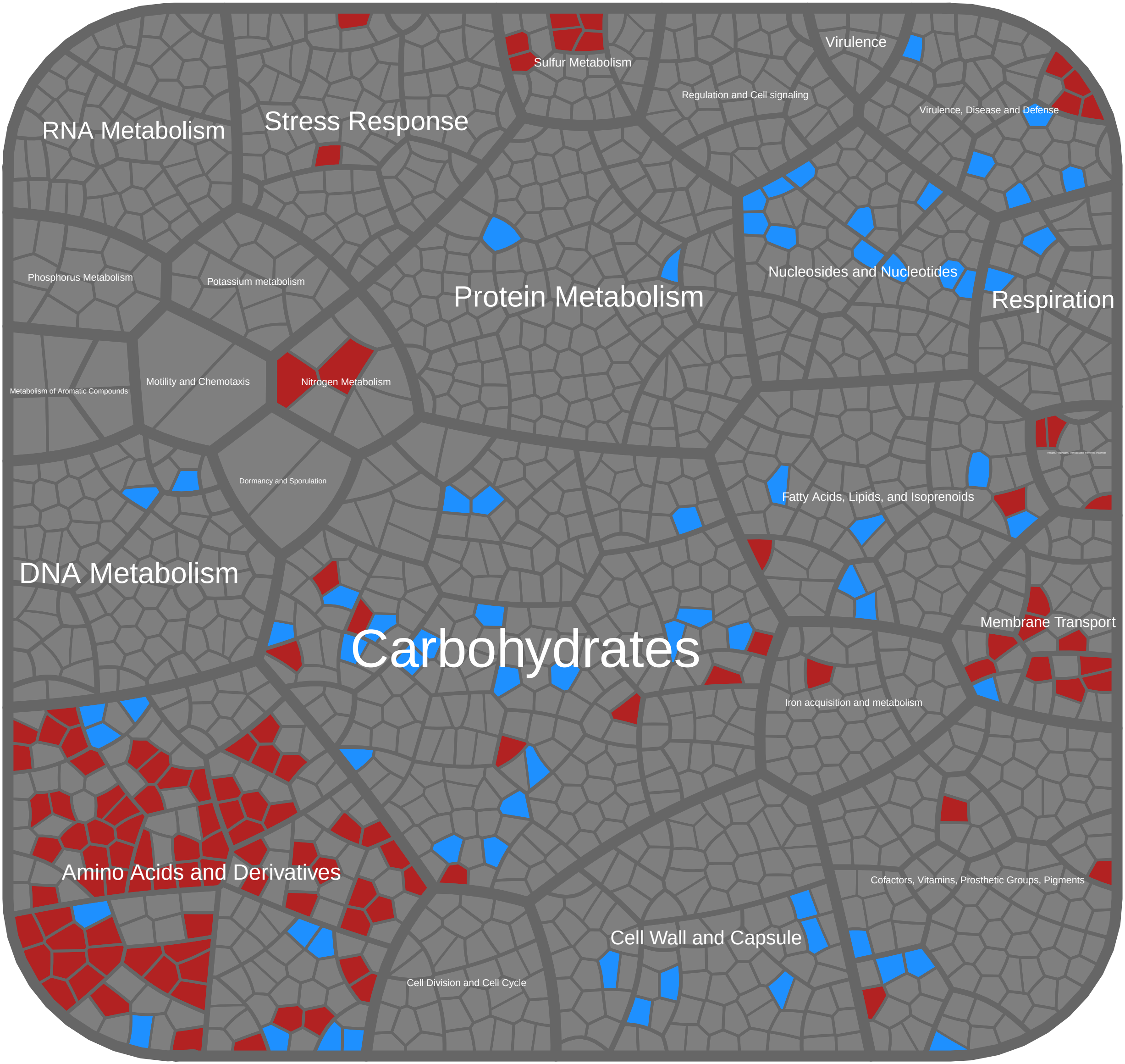
Virulence
Sulfur Metabolism
Regulation and Cell signaling
Virulence, Disease and Defense
RNA Metabolism
Stress Response
Phosphorus Metabolism
Potassium metabolism
Protein Metabolism
Nucleosides and Nucleotides
Respiration
Metabolism of Aromatic Compounds
Motility and Chemotaxis
Nitrogen Metabolism
Dormancy and Sporulation
Fatty Acids, Lipids, and Isoprenoids
DNA Metabolism
Membrane Transport
Carbohydrates
Iron acquisition and metabolism
Amino Acids and Derivatives
Cofactors, Vitamins, Prosthetic Groups, Pigments
Cell Wall and Capsule
Cell Division and Cell Cycle

saHPF_exp

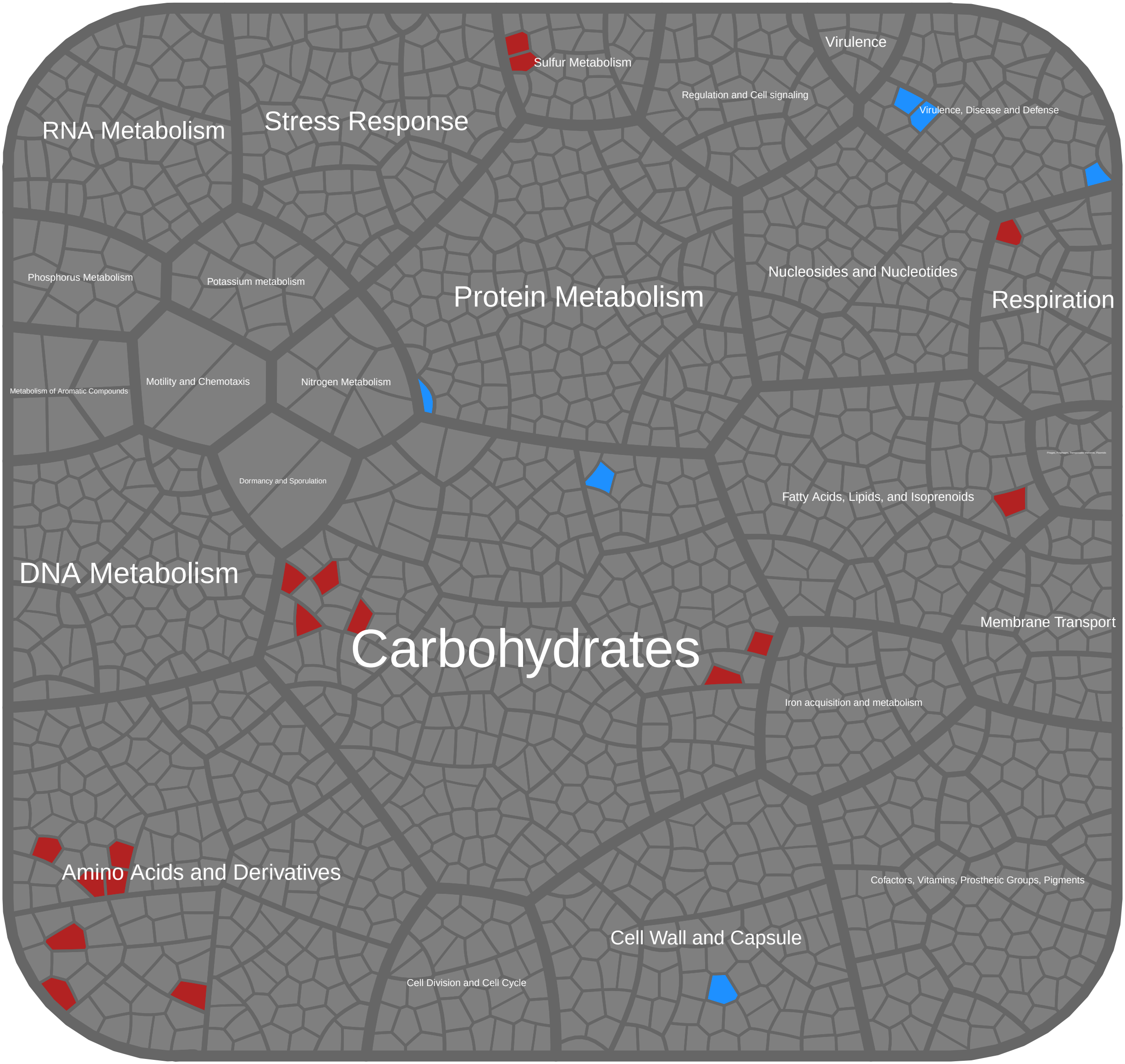

Sulfur Metabolism
Virulence
Regulation and Cell signaling
Virulence, Disease and Defense
RNA Metabolism
Stress Response
Phosphorus Metabolism
Potassium metabolism
Protein Metabolism
Nucleosides and Nucleotides
Respiration
Metabolism of Aromatic Compounds
Motility and Chemotaxis
Nitrogen Metabolism
Dormancy and Sporulation
Fatty Acids, Lipids, and Isoprenoids
DNA Metabolism
Membrane Transport
Carbohydrates
Iron acquisition and metabolism
Amino Acids and Derivatives
Cofactors, Vitamins, Prosthetic Groups, Pigments
Cell Wall and Capsule
Cell Division and Cell Cycle

saHPF_stat

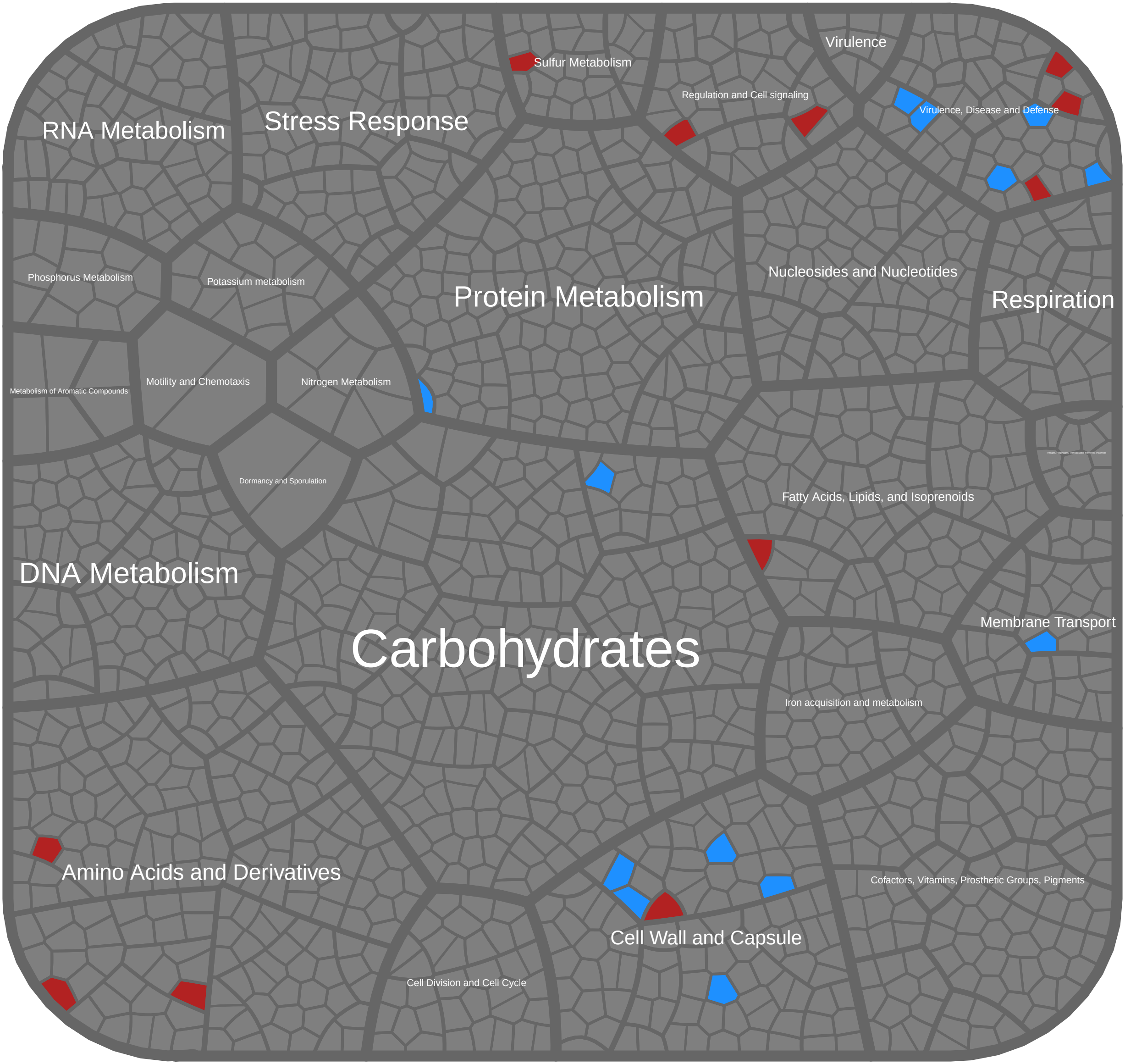
Sulfur Metabolism
Virulence
Regulation and Cell signaling
Virulence, Disease and Defense
RNA Metabolism
Stress Response
Phosphorus Metabolism
Potassium metabolism
Protein Metabolism
Nucleosides and Nucleotides
Respiration
Metabolism of Aromatic Compounds
Motility and Chemotaxis
Nitrogen Metabolism
Dormancy and Sporulation
Fatty Acids, Lipids, and Isoprenoids
DNA Metabolism
Membrane Transport
Carbohydrates
Iron acquisition and metabolism
Amino Acids and Derivatives
Cofactors, Vitamins, Prosthetic Groups, Pigments
Cell Wall and Capsule
Cell Division and Cell Cycle

sigB_exp

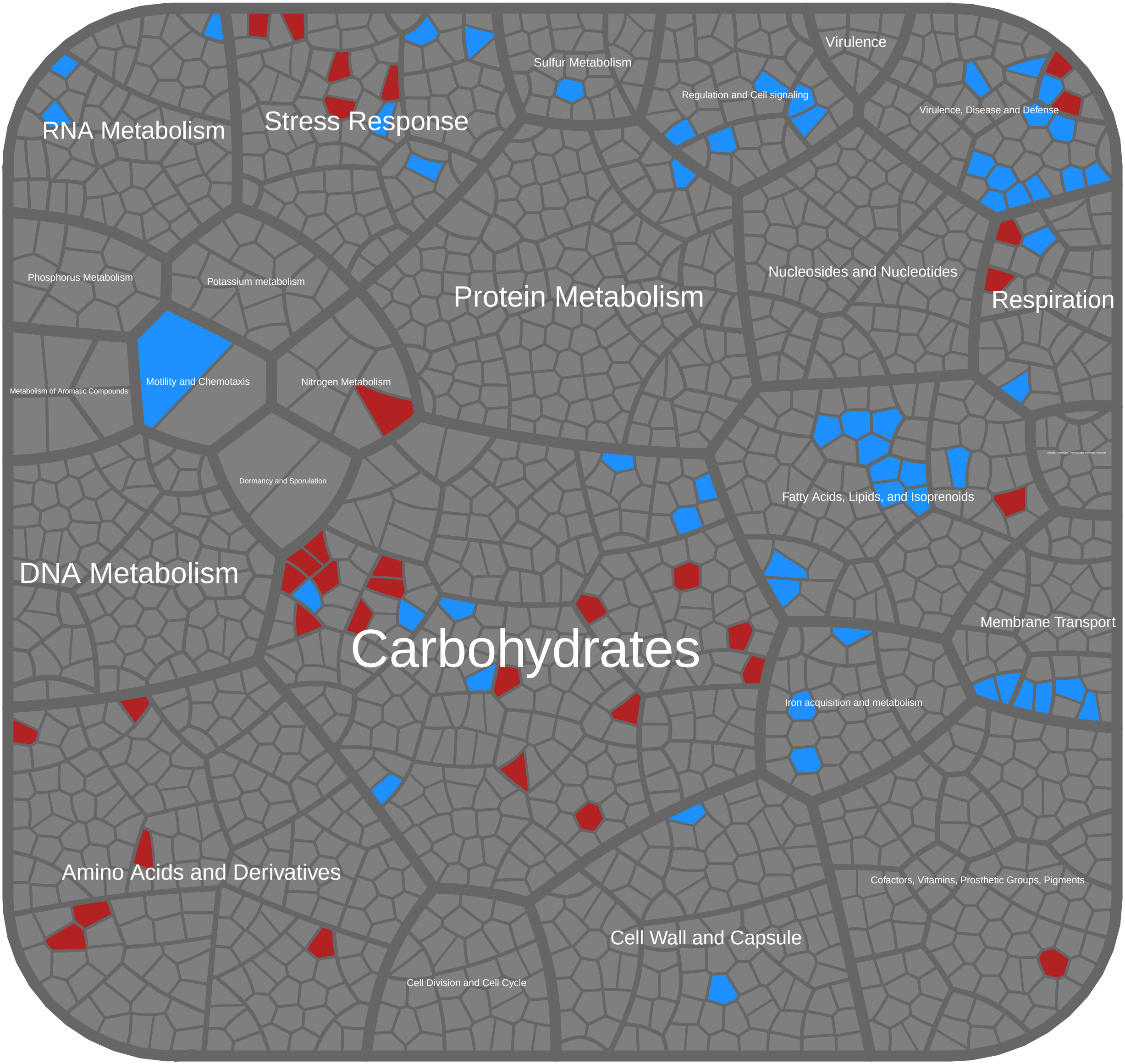

RNA Metabolism
Stress Response
Sulfur Metabolism
Virulence
Regulation and Cell signaling
Virulence, Disease and Defense
Phosphorus Metabolism
Potassium metabolism
Protein Metabolism
Nucleosides and Nucleotides
Respiration
Metabolism of Aromatic Compounds
Motility and Chemotaxis
Nitrogen Metabolism
Dormancy and Sporulation
Fatty Acids, Lipids, and Isoprenoids
DNA Metabolism
Membrane Transport
Carbohydrates
Iron acquisition and metabolism
Amino Acids and Derivatives
Cofactors, Vitamins, Prosthetic Groups, Pigments
Cell Wall and Capsule
Cell Division and Cell Cycle

sigB_stat

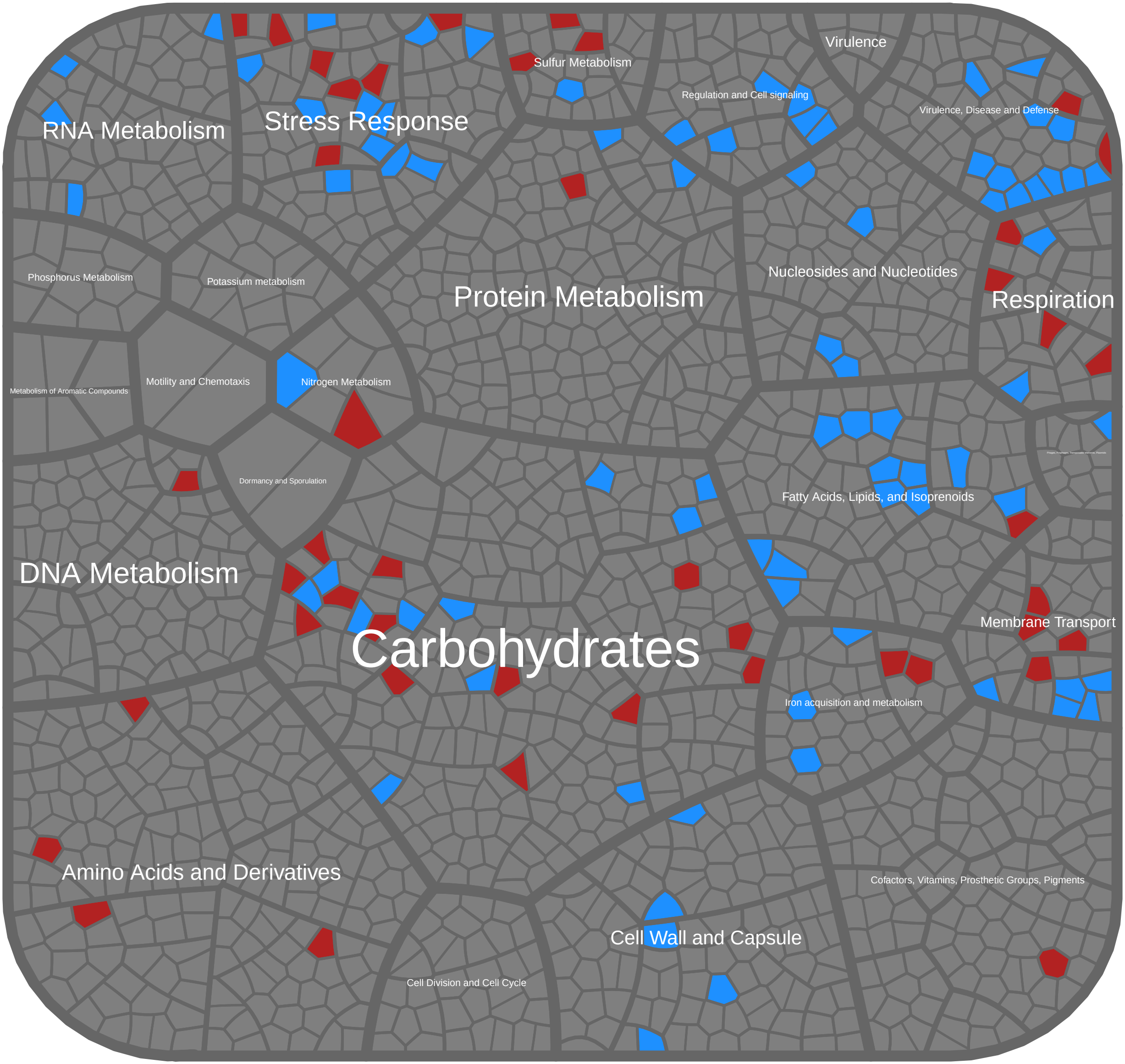
Virulence
Sulfur Metabolism
Regulation and Cell signaling
Virulence, Disease and Defense
RNA Metabolism
Stress Response
Phosphorus Metabolism
Potassium metabolism
Protein Metabolism
Nucleosides and Nucleotides
Respiration
Metabolism of Aromatic Compounds
Motility and Chemotaxis
Nitrogen Metabolism
Dormancy and Sporulation
Fatty Acids, Lipids, and Isoprenoids
DNA Metabolism
Membrane Transport
Carbohydrates
Iron acquisition and metabolism
Amino Acids and Derivatives
Cofactors, Vitamins, Prosthetic Groups, Pigments
Cell Wall and Capsule
Cell Division and Cell Cycle

Figure S3. Voronoi treemaps representing the functions and relative abundance of cellular proteins of the *sigB*, *codY* or *saHPF* mutant bacteria. The known functional categories according to TheSEED of identified proteins are visualized as Voronoi treemaps taking into account all functional categories. The size of each functional category is proportional to the number of identified proteins belonging to the respective functional category. Red represents proteins that were detected as significantly elevated compared to the USA300 WT strain, blue represents proteins detected as significantly reduced and grey represents proteins with no significant difference in abundance. The different treemaps show the relative abundances of cellular proteins from *codY*, *saHPF*, or *sigB* mutant bacteria in the exponential (exp) or stationary (stat) growth phases. Note that the Voronoi treemaps are presented in a separate pdf file.

Figure S4 Global overview of differentially abundant proteins

compared to USA300

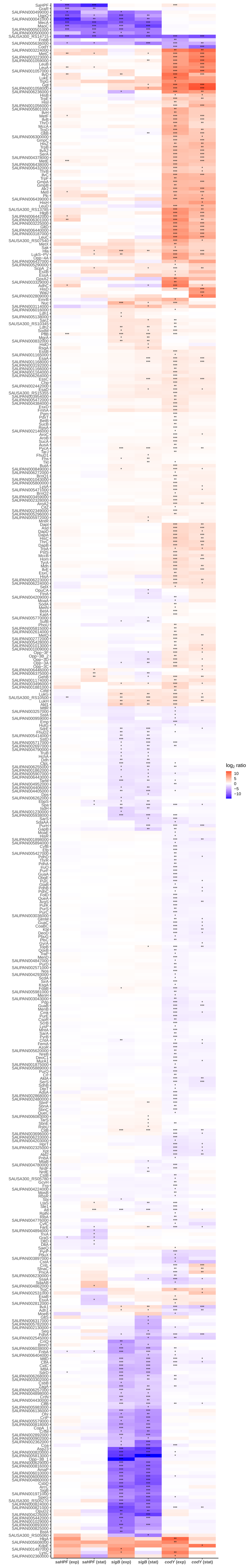

*: p.fdr < 0.05; **: p.fdr < 0.01; ***: p.fdr < 0.001

Figure S4. Heatmap showing the relative abundance of cellular proteins of which the levels were significantly changed in at least one of the *saHPF*, *sigB*, or *codY* mutant bacterial strains compared to the USA300 WT bacteria in the exponential (exp) or stationary (stat) growth phases. The relative abundance of the identified proteins was determined based on the median of peptide intensities using the PECA package, and it is indicated by color-coded bars. Names of the different proteins are indicated on the left side of the heatmap and a legend for the color code on the right side. Note that the heatmap is presented as a separate pdf file.

Figure S5

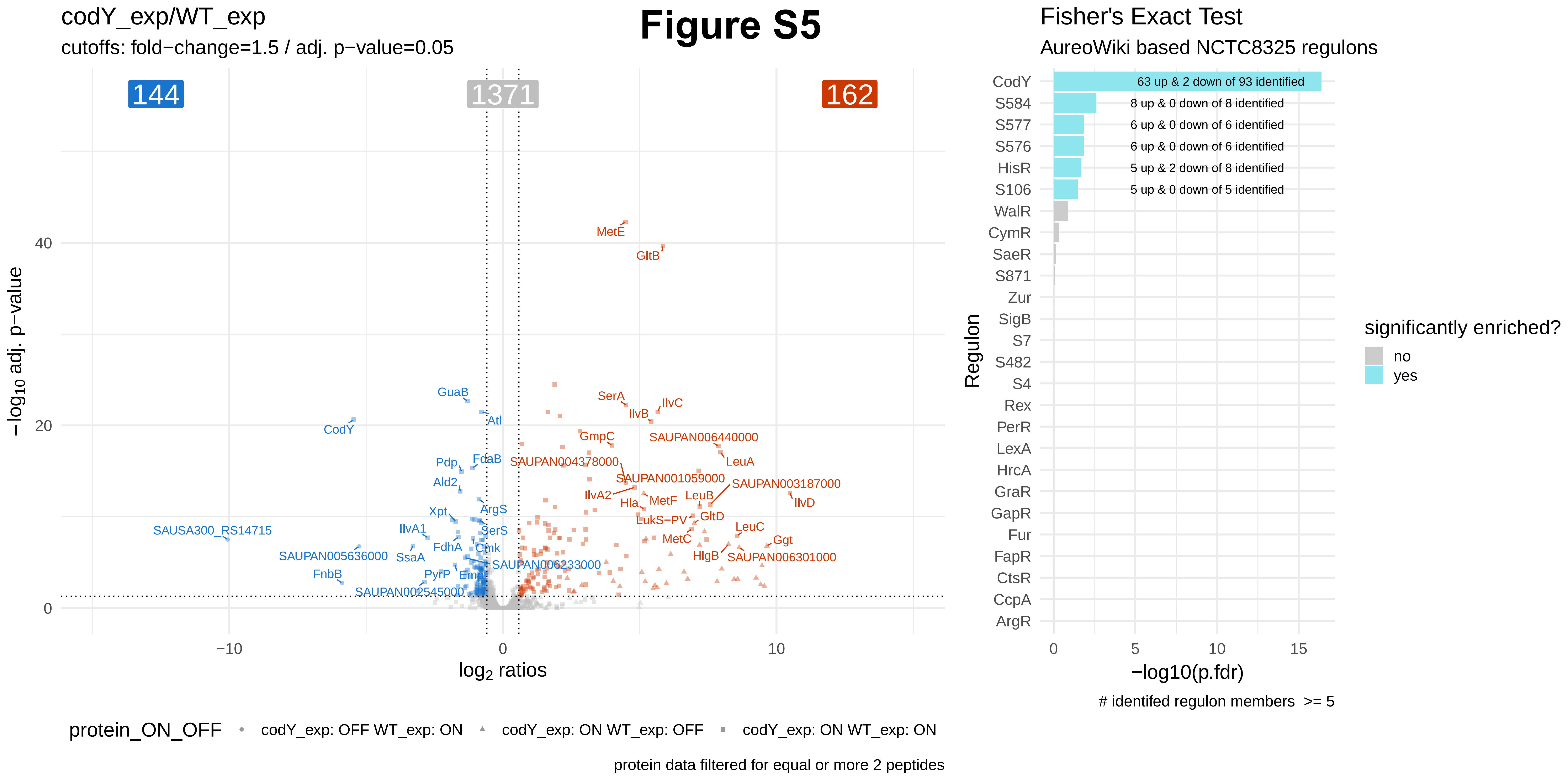

codY_exp/WT_exp
cutoffs: fold−change=1.5 / adj. p−value=0.05
144
1371
162
MetE
GltB
GuaB
Atl
CodY
SerA
IlvC
IlvB
GmpC
SAUPAN006440000
LeuA
Pdp
FdaB
SAUPAN004378000
Ald2
SAUPAN001059000
SAUPAN003187000
IlvA2
Hla
MetF
LeuB
IlvD
Xpt
ArgS
LukS−PV
GltD
SAUSA300_RS14715
IlvA1
SerS
LeuC
FdhA
Cmk
MetC
Ggt
SAUPAN005636000
SsaA
HlgB
SAUPAN006301000
SAUPAN006233000
FnbB
PyrP
Emp
SAUPAN002545000
−log10 adj. p−value
log2 ratios
protein_ON_OFF
codY_exp: OFF WT_exp: ON
codY_exp: ON WT_exp: OFF
codY_exp: ON WT_exp: ON
protein data filtered for equal or more 2 peptides
Fisher's Exact Test
AureoWiki based NCTC8325 regulons
63 up & 2 down of 93 identified
8 up & 0 down of 8 identified
6 up & 0 down of 6 identified
6 up & 0 down of 6 identified
5 up & 2 down of 8 identified
5 up & 0 down of 5 identified
CodY
S584
S577
S576
HisR
S106
WalR
CymR
SaeR
S871
Zur
SigB
S7
S482
S4
Rex
PerR
LexA
HrcA
GraR
GapR
Fur
FapR
CtsR
CcpA
ArgR
Regulon
−log10(p.fdr)
identifed regulon members >= 5
significantly enriched?
no
yes

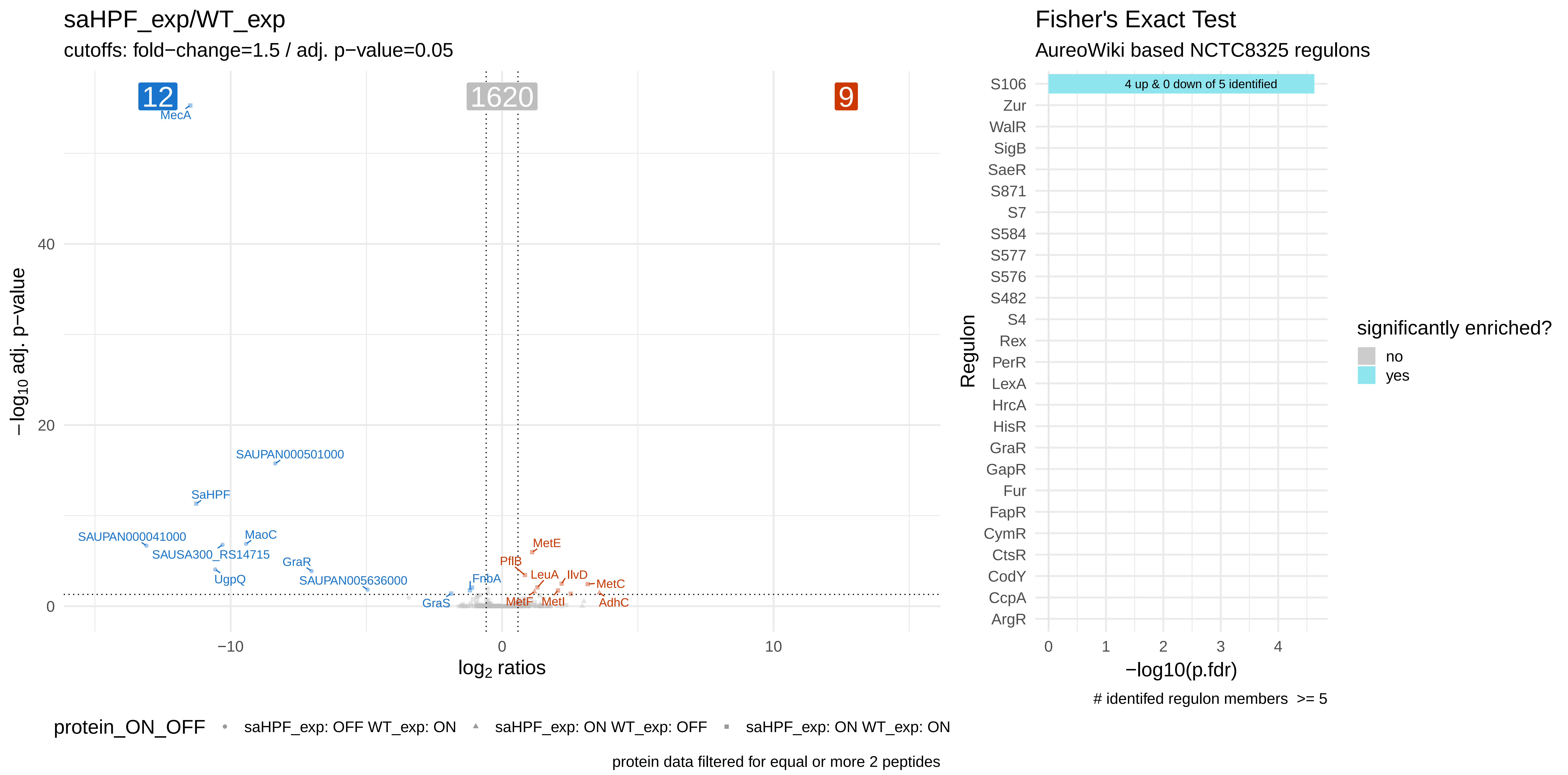

saHPF_exp/WT_exp
cutoffs: fold−change=1.5 / adj. p−value=0.05
12
1620
9
MecA
SAUPAN000501000
SaHPF
SAUPAN000041000
MaoC
SAUSA300_RS14715
GraR
UgpQ
SAUPAN005636000
FnbA
GraS
MetE
PflB
LeuA
IlvD
MetC
MetF
MetI
AdhC
−log10 adj. p−value
40
20
0
−10
0
10
log2 ratios
protein_ON_OFF
saHPF_exp: OFF WT_exp: ON
saHPF_exp: ON WT_exp: OFF
saHPF_exp: ON WT_exp: ON
protein data filtered for equal or more 2 peptides
Fisher's Exact Test
AureoWiki based NCTC8325 regulons
S106
4 up & 0 down of 5 identified
Zur
WalR
SigB
SaeR
S871
S7
S584
S577
S576
S482
S4
Rex
PerR
LexA
HrcA
HisR
GraR
GapR
Fur
FapR
CymR
CtsR
CodY
CcpA
ArgR
Regulon
0
1
2
3
4
−log10(p.fdr)
identifed regulon members >= 5
significantly enriched?
no
yes

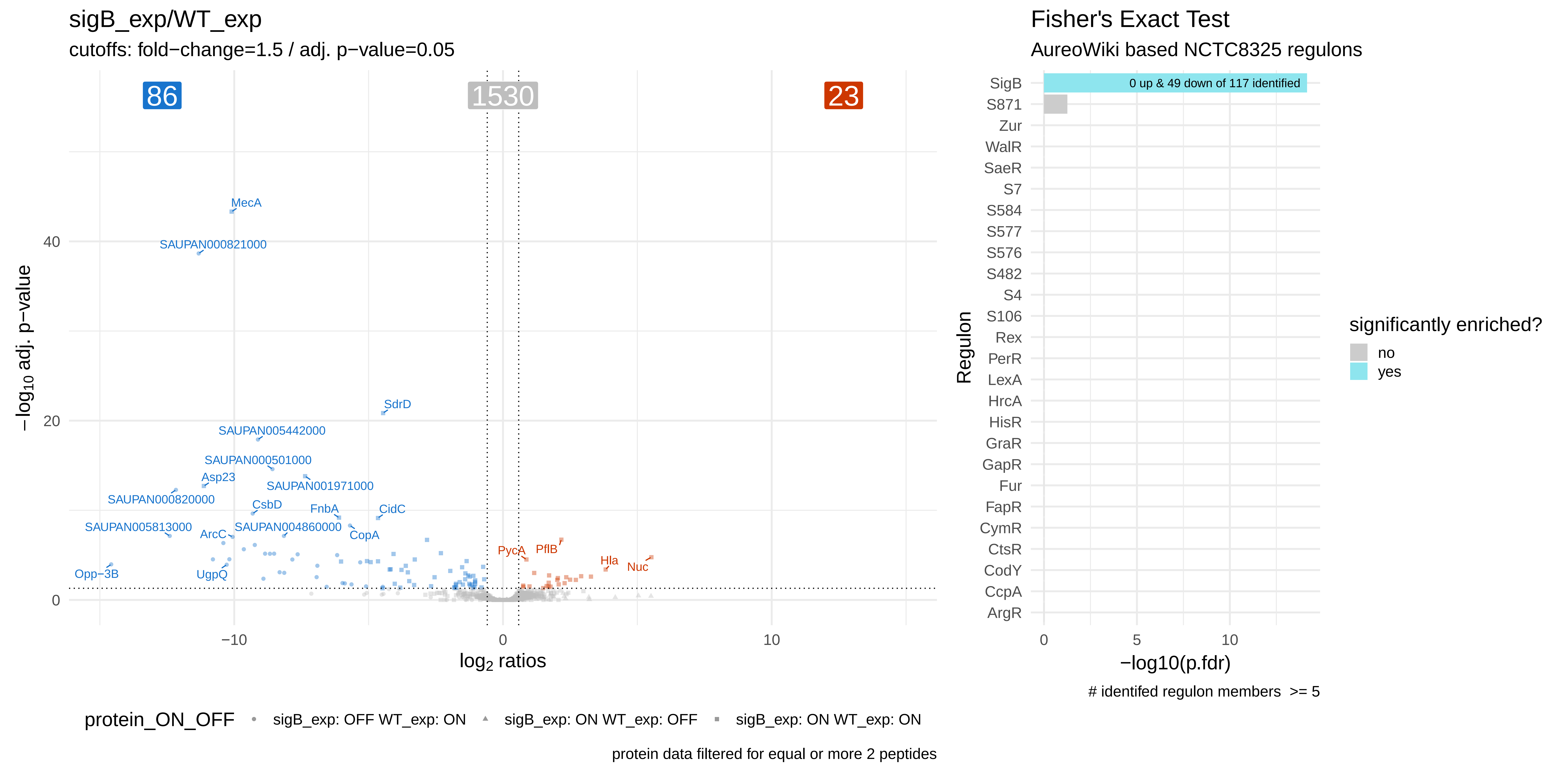
sigB_exp/WT_exp
cutoffs: fold−change=1.5 / adj. p−value=0.05
86
1530
23
MecA
SAUPAN000821000
SdrD
SAUPAN005442000
SAUPAN000501000
Asp23
SAUPAN001971000
SAUPAN000820000
CsbD
FnbA
CidC
SAUPAN005813000
ArcC
SAUPAN004860000
CopA
PycA
PflB
Hla
Nuc
Opp−3B
UgpQ
−log10 adj. p−value
log2 ratios
protein_ON_OFF
sigB_exp: OFF WT_exp: ON
sigB_exp: ON WT_exp: OFF
sigB_exp: ON WT_exp: ON
protein data filtered for equal or more 2 peptides
Fisher's Exact Test
AureoWiki based NCTC8325 regulons
0 up & 49 down of 117 identified
SigB
S871
Zur
WalR
SaeR
S7
S584
S577
S576
S482
S4
S106
Rex
PerR
LexA
HrcA
HisR
GraR
GapR
Fur
FapR
CymR
CtsR
CodY
CcpA
ArgR
Regulon
−log10(p.fdr)
identifed regulon members >= 5
significantly enriched?
no
yes

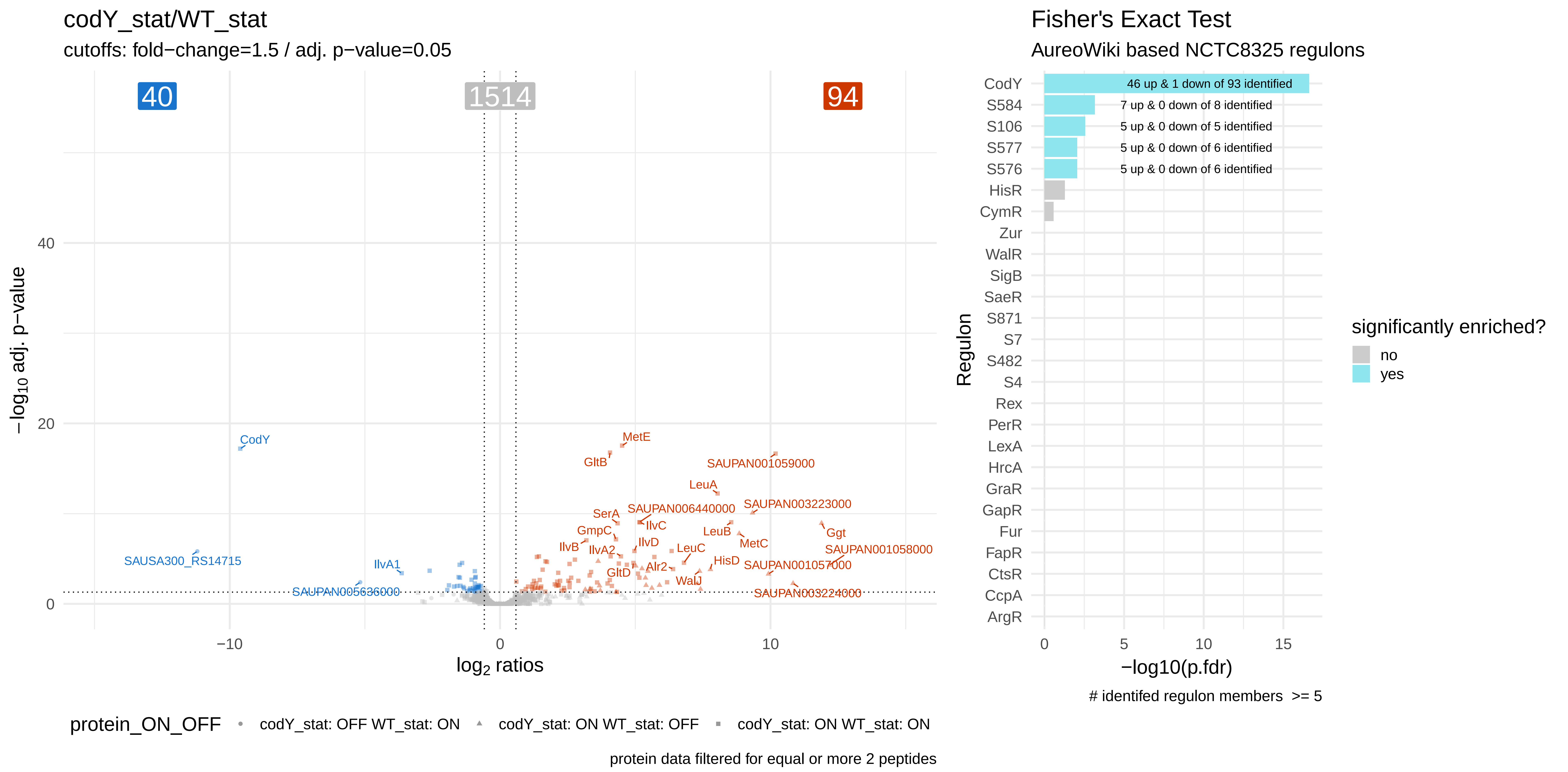

codY_stat/WT_stat
cutoffs: fold−change=1.5 / adj. p−value=0.05
40
1514
94
CodY
SAUSA300_RS14715
IlvA1
SAUPAN005636000
MetE
GltB
SAUPAN001059000
LeuA
SerA
SAUPAN006440000
SAUPAN003223000
GmpC
IlvC
LeuB
Ggt
IlvB
IlvA2
IlvD
LeuC
MetC
SAUPAN001058000
GltD
Alr2
HisD
SAUPAN001057000
WalJ
SAUPAN003224000
−log10 adj. p−value
log2 ratios
protein_ON_OFF
codY_stat: OFF WT_stat: ON
codY_stat: ON WT_stat: OFF
codY_stat: ON WT_stat: ON
protein data filtered for equal or more 2 peptides
Fisher's Exact Test
AureoWiki based NCTC8325 regulons
46 up & 1 down of 93 identified
7 up & 0 down of 8 identified
5 up & 0 down of 5 identified
5 up & 0 down of 6 identified
5 up & 0 down of 6 identified
Regulon
CodY
S584
S106
S577
S576
HisR
CymR
Zur
WalR
SigB
SaeR
S871
S7
S482
S4
Rex
PerR
LexA
HrcA
GraR
GapR
Fur
FapR
CtsR
CcpA
ArgR
−log10(p.fdr)
identifed regulon members >= 5
significantly enriched?
no
yes

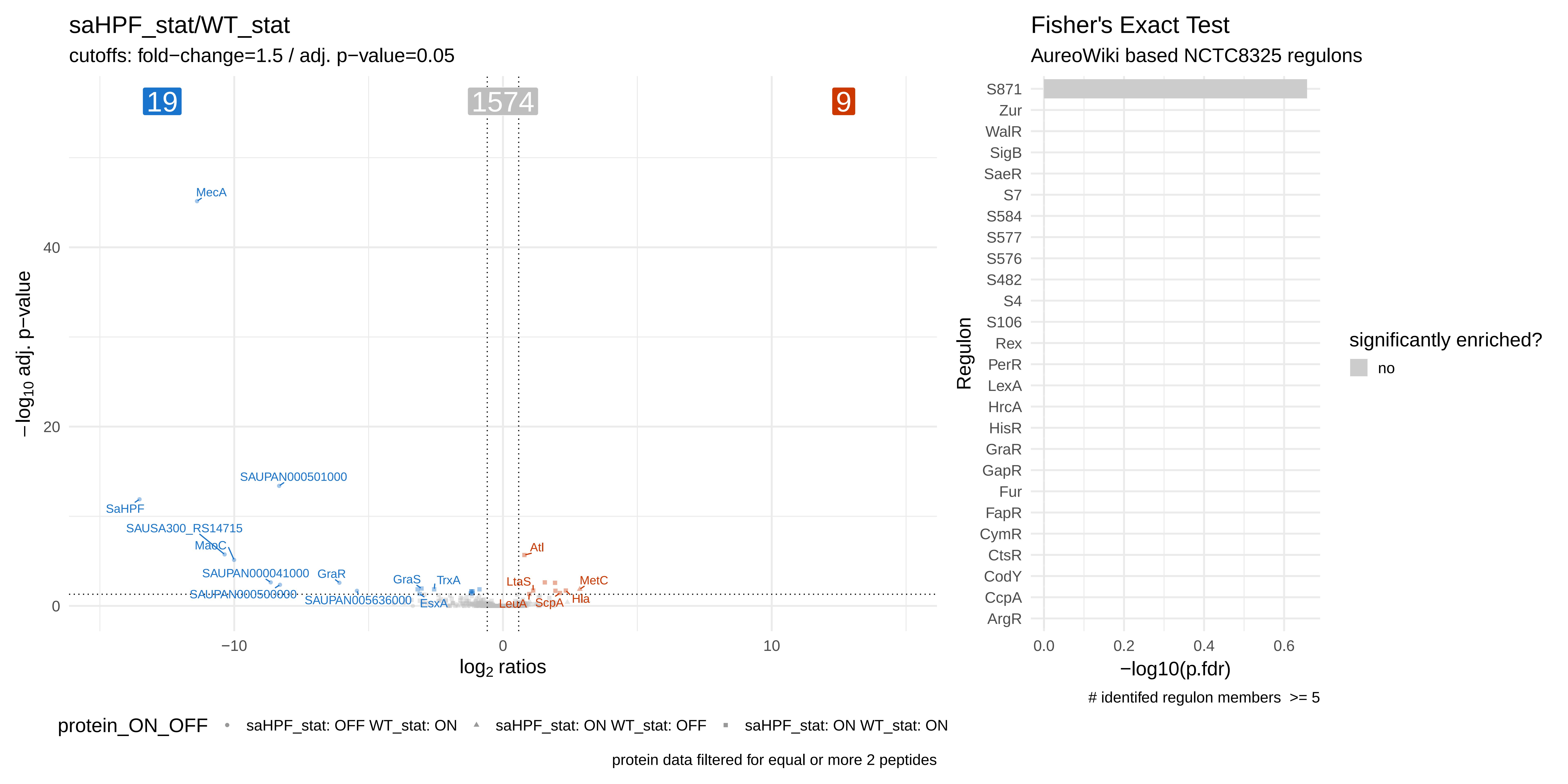
saHPF_stat/WT_stat
cutoffs: fold−change=1.5 / adj. p−value=0.05
19
1574
9
MecA
SAUPAN000501000
SaHPF
SAUSA300_RS14715
MaoC
SAUPAN000041000
GraR
SAUPAN000500000
SAUPAN005636000
GraS
TrxA
EsxA
Atl
LtaS
MetC
LeuA
ScpA
Hla
40
20
0
−10
0
10
−log10 adj. p−value
log2 ratios
protein_ON_OFF
saHPF_stat: OFF WT_stat: ON
saHPF_stat: ON WT_stat: OFF
saHPF_stat: ON WT_stat: ON
protein data filtered for equal or more 2 peptides
Fisher's Exact Test
AureoWiki based NCTC8325 regulons
S871
Zur
WalR
SigB
SaeR
S7
S584
S577
S576
S482
S4
S106
Rex
PerR
LexA
HrcA
HisR
GraR
GapR
Fur
FapR
CymR
CtsR
CodY
CcpA
ArgR
Regulon
0.0
0.2
0.4
0.6
−log10(p.fdr)
identifed regulon members >= 5
significantly enriched?
no

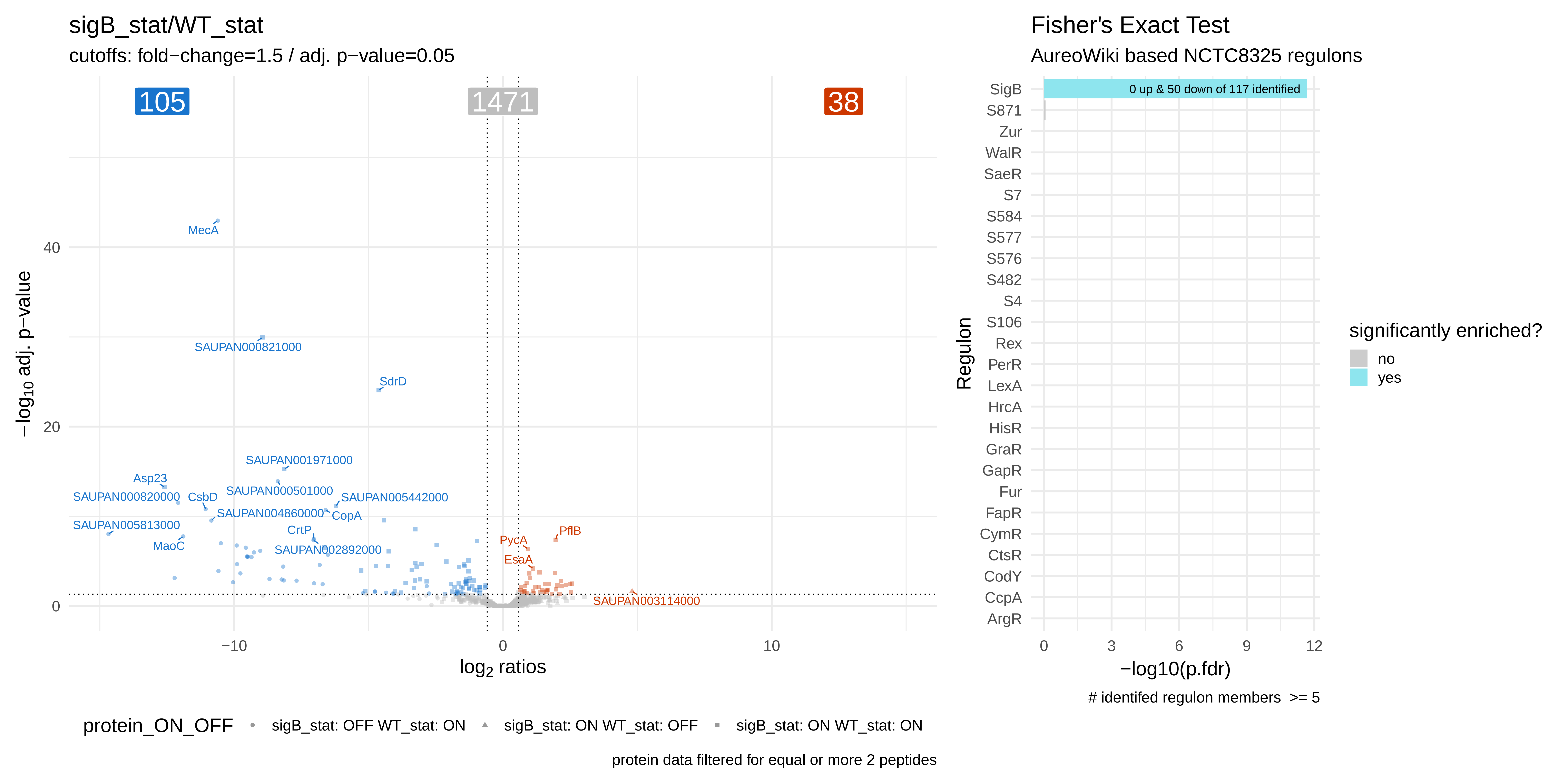
sigB_stat/WT_stat
cutoffs: fold−change=1.5 / adj. p−value=0.05
105
1471
38
MecA
SAUPAN000821000
SdrD
SAUPAN001971000
Asp23
SAUPAN000501000
SAUPAN000820000
CsbD
SAUPAN005442000
SAUPAN004860000
CopA
SAUPAN005813000
CrtP
MaoC
SAUPAN002892000
PycA
PflB
EsaA
SAUPAN003114000
−log10 adj. p−value
log2 ratios
protein_ON_OFF
sigB_stat: OFF WT_stat: ON
sigB_stat: ON WT_stat: OFF
sigB_stat: ON WT_stat: ON
protein data filtered for equal or more 2 peptides
Fisher's Exact Test
AureoWiki based NCTC8325 regulons
0 up & 50 down of 117 identified
SigB
S871
Zur
WalR
SaeR
S7
S584
S577
S576
S482
S4
S106
Rex
PerR
LexA
HrcA
HisR
GraR
GapR
Fur
FapR
CymR
CtsR
CodY
CcpA
ArgR
Regulon
−log10(p.fdr)
identifed regulon members >= 5
significantly enriched?
no
yes

Figure S5. Volcano plots and Fisher's exact tests showing significantly different protein abundances in the *codY*, *sigB* or *saHPF* mutant bacteria and the USA300 WT bacteria. Individual dots in the volcano plots mark statistically different abundances for the indicated proteins. The top 25 proteins are labeled. The table with the outcomes of the Fisher exact tests summarize the numbers of proteins identified at statistically significantly elevated ('up') or reduced ('down') proteins per regulon. Note that the Volcano plots and Fisher's exact tests are presented in a separate pdf file.
