# Supplementary material for: Functional and Proteomic Dissection of the Contributions of CodY, SigB and the Hibernation Promoting Factor HPF to Interactions of Staphylococcus aureus USA300 with Human Lung Epithelial Cells
Source: J Proteome Res. 2024 Sep 20;23(10):4742–60. doi: 10.1021/acs.jproteome.4c00724 (PMC11459534; doi:10.1021/acs.jproteome.4c00724)
Supplement: Supplementary file 2 — pr4c00724_si_002.pdf [file pr4c00724_si_002.pdf]

Supplemental Tables S1 and S4

Functional and proteomic dissection of the contributions of CodY, SigB and the hibernation promoting factor HPF to interactions of *Staphylococcus aureus* USA300 with human lung epithelial cells

Xiaofang Li[1], Larissa M. Busch[2], Sjouke Piersma[1], Min Wang[1], Lei Liu[1], Manuela Gesell Salazar[2], Kristin Surmann[2], Ulrike Mäder[2], Uwe Völker[2], Girbe Buist[1#] and Jan Maarten van Dijl[1#*],

[1]Department of Medical Microbiology and Infection Prevention, University of Groningen, University Medical Center Groningen, Hanzeplein 1, 9700 RB Groningen, the Netherlands

[2]Interfaculty Institute for Genetics and Functional Genomics, Department Functional Genomics, University Medicine Greifswald, D-17475 Greifswald, Germany

[*]**Corresponding author**: Jan Maarten van Dijl, University of Groningen, University Medical Center Groningen, Hanzeplein 1, 9700 RB Groningen, the Netherlands, tel. +31-50-3615187, e-mail: j.m.van.dijl01@umcg.nl

[#]Equal contributions

Table S1 A. Parameters for reversed phase liquid chromatography (RPLC).

instrument	**Ultimate 3000 RSLC (Thermo Scientific)**
trap column	75 µm inner diameter, packed with 3 µm C18 particles (Acclaim PepMap100, Thermo Scientific)
analytical column	Accucore 150-C18, (Thermo Fisher Scientific)<br>25 cm x 75 µm, 2,6 µm C18 particles, 150 Å pore size
buffer system	binary buffer system consisting of 0.1% acetic acid in HPLC-grade water (buffer A) and 100% ACN in 0.1% acetic acid (buffer B)
flow rate	300 nl/min
gradient	linear gradient of buffer B from 5% up to 25% in 120 min<br>0 min: 2%B<br>2min 5% B<br>10min 5% B<br>130 min: 25%B<br>135 min 40 %B<br>137 min 90 %B<br>142 min 90 %B<br>145 min 2 %B<br>150 min 2 %B
column oven temperature	40°C

Table S1 B. Parameters for mass spectrometry analysis in data-independent mode.

instrument	*Q Exactive HF*
electrospray	Nanospray Flex Ion Source
operation mode	data-independent
Full MS	
Polarity	positive
MS scan resolution	60000
AGC target	5e6
maximum ion injection time for the MS scan	200 ms
Scan range	333 to 1650 m/z

Spectra data type	profile
dd-MS2	
Polarity	positive
Resolution	30,000
MS/MS AGC target	3e6
maximum ion injection time for the MS/MS scans	auto
Spectra data type	profile
Microscans	1
isolation window	56 windows, 13 m/z
Fixed first mass	-
dissociation mode	higher energy collisional dissociation (HCD)
normalized collision energy	27.5%

Table S1 C. Spectronaut parameters used for analysis of mass spectrometry data.

Parameter	**Setting**
Identification	
Pvalue Estimator	Kernel Density Estimator
Precursor Qvalue Cutoff	0.001
Quantification	
Precursor Filtering	Identified (Qvalue)
Imputation Strategy	Use Background Signal
Quantity MS Level	MS2
Quantity Type	Area
Cross-Run Normalization	True
Normalization Strategy	Global Normalization (Median)
Row Selection	Identified in at least 1 Run (Sparse)

Table S1 D. R packages used for data analysis and visualization.

**PACKAGE**	**VERSION**	**REFERENCE**
tidyverse	1.3.1	(1)

openxlsx	4.2.5	(2)
readxl	1.4.0	(3)
readr	2.1.2	(4)
FactoMineR	2.4	(5)
iq	1.9.6	(6)
PECA	1.30.0	(7)
ggrepel	0.9.1	(8)
patchwork	1.1.1	(9)
scales	1.2.0	(10)
vroom	1.5.7	(11)
WeightedTreemaps	0.1.1	(12)
ggtext	0.1.2	(13)

(1) Wickham et al., (2019). Welcome to the tidyverse. Journal of Open Source Software, 4(43), 1686, https://doi.org/10.21105/joss.01686

(2) Philipp Schauberger and Alexander Walker (2021). openxlsx: Read, Write and Edit xlsx Files. R package version 4.2.5. https://CRAN.R-project.org/package=openxlsx

(3) Hadley Wickham and Jennifer Bryan (2022). readxl: Read Excel Files. R package version 1.4.0. https://CRAN.R-project.org/package=readxl

(4) Hadley Wickham, Jim Hester and Jennifer Bryan (2022). readr: Read Rectangular Text Data. R package version 2.1.2. https://CRAN.R-project.org/package=readr

(5) Sebastien Le, Julie Josse, Francois Husson (2008). FactoMineR: An R Package for Multivariate Analysis. Journal of Statistical Software, 25(1), 1-18. 10.18637/jss.v025.i01

(6) Pham T, Henneman A, Jimenez C (2020). “iq: an R package to estimate relative protein abundances from ion quantification in DIA-MS-based proteomics.” _Bioinformatics_, *36*(8), 2611-2613. <URL:

https://doi.org/10.1093/bioinformatics/btz961>.

(7) Tomi Suomi, Jukka Hiissa and Laura L. Elo (2021). PECA: Probe-level Expression Change Averaging. R package version 1.30.0.

(8) Kamil Slowikowski (2021). ggrepel: Automatically Position Non-Overlapping Text Labels with 'ggplot2'. R package version 0.9.1. https://CRAN.R-project.org/package=ggrepel

(9) Thomas Lin Pedersen (2020). patchwork: The Composer of Plots. R package version 1.1.1. https://CRAN.R-project.org/package=patchwork

(10) Hadley Wickham and Dana Seidel (2022). scales: Scale Functions for Visualization. R package version 1.2.0. https://CRAN.R-project.org/package=scales

(11) Jim Hester, Hadley Wickham and Jennifer Bryan (2021). vroom: Read and Write Rectangular Text Data Quickly. R package version 1.5.7. https://CRAN.R-project.org/package=vroom

(12) https://github.com/m-jahn/WeightedTreemaps

(13) Claus O. Wilke and Brenton M. Wiernik (2022). ggtext: Improved Text Rendering Support for 'ggplot2'. R package version 0.1.2. https://CRAN.R-project.org/package=ggtext

Table S4. Summary and list of functional protein annotations in virulence, antimicrobial resistance, adhesion to host cells and cytotoxicity of the *codY, sigB* or *saHPF* mutant bacteria, relative to USA300 WT bacteria.

Comparison vs. USA300	virulence			Antimicrobial resistance			Adhesion to cells			cytotoxicity		
	down	none	up	down	none	up	down	none	up	down	none	up
codY (exp)	7	49	11	0	15	0	7	12	0	0	1	4
codY (stat)	4	55	8	0	15	0	4	15	0	0	1	4
saHPF (exp)	1	66	0	0	15	0	1	18	0	0	5	0
saHPF (stat)	3	60	4	0	15	0	3	15	1	0	3	2
sigB (exp)	11	51	5	0	15	0	9	10	0	1	2	2
sigB (stat)	12	51	4	0	15	0	10	9	0	0	4	1
